# Evaluation of the antitumor effects of PP242 in a colon cancer xenograft mouse model using comprehensive metabolomics and lipidomics

**DOI:** 10.1038/s41598-020-73721-w

**Published:** 2020-10-16

**Authors:** Md Mamunur Rashid, Hyunbeom Lee, Byung Hwa Jung

**Affiliations:** 1 Molecular Recognition Research Center, Korea Institute of Science and Technology, Seoul, 02792 South Korea; 2 Division of Bio-Medical Science and Technology, KIST School, Korea University of Science and Technology (UST), Seoul, 02792 South Korea

**Keywords:** Mass spectrometry, Metabolomics

## Abstract

PP242, an inhibitor of mechanistic target of rapamycin (mTOR), displays potent anticancer effects against various cancer types. However, the underlying metabolic mechanism associated with the PP242 effects is not clearly understood. In this study, comprehensive metabolomics and lipidomics investigations were performed using ultra-high-performance chromatography-Orbitrap-mass spectrometry (UHPLC-Orbitrap-MS) in plasma and tumor tissue to reveal the metabolic mechanism of PP242 in an LS174T cell-induced colon cancer xenograft mouse model. After 3 weeks of PP242 treatment, a reduction in tumor size and weight was observed without any critical toxicities. According to results, metabolic changes due to the effects of PP242 were not significant in plasma. In contrast, metabolic changes in tumor tissues were very significant in the PP242-treated group compared to the xenograft control (XC) group, and revealed that energy and lipid metabolism were mainly altered by PP242 treatment like other cancer inhibitors. Additionally, in this study, it was discovered that not only TCA cycle but also fatty acid β-oxidation (β-FAO) for energy metabolism was inhibited and clear reduction in glycerophospholipid was observed. This study reveals new insights into the underlying anticancer mechanism of the dual mTOR inhibitor PP242, and could help further to facilitate the understanding of PP242 effects in the clinical application.

## Introduction

Colon cancer also known as colorectal cancer (CRC) or bowel cancer, is the fourth most common cancer and the third leading cause of cancer-related deaths among all cancers worldwide^1^. The progression of CRC is associated with multiple genetic alterations, such as the activation of Ras, the overactivation of the phosphatidylinositol 3-kinase pathway, mutation of p53, and Wnt pathway dysregulation^2^. In addition, emerging data have implicated frequent activation of mTOR with the progression of most cancers, including CRC^3,4^, which represents mTOR as a compelling therapeutic target for cancer treatment.

The mammalian or mechanistic target of rapamycin (mTOR) is a serine/threonine protein kinase that regulates many cellular functions, such as protein synthesis, cell proliferation, growth, survival, metabolism, autophagy and senescence, through two distinct multiprotein complexes named mTOR complex 1 (mTORC1) and mTOR complex 2 (mTORC2)^5,6^. mTOR regulates normal cellular functions through integrating signals from its upstream proteins PI3K and AKT (the PI3K/AKT/mTOR pathway). Dysregulation of this pathway led to the development of several fatal diseases, including metabolic, cardiovascular, neurological, and immunological diseases and cancers^7^. Since the mTOR pathway is dysregulated in many cancers, inhibition of mTOR represents a compelling therapeutic target for cancer treatment.

Depending on the inhibitory characteristics of mTOR complexes (mTORC1 and mTORC2), mTOR inhibitors are classified into two generations. Rapamycin and its analogs are considered first-generation mTOR inhibitors due to their ability to inhibit only mTORC1 activity^8^. Although prolonged exposure to rapamycin can also partially inhibit mTORC2, the inhibition is transient and not efficient for cancer treatment^9^. Therefore, efforts have been made to develop second-generation mTOR inhibitors that can efficiently inhibit both mTOR complexes. To date, numerous second-generation mTOR inhibitors have been developed to efficiently treat cancers. PP242 is an ATP-competitive second-generation inhibitor of mTOR that exerts its anticancer activity on numerous types of cancer. Previous studies have reported that PP242 can inhibit the proliferation, migration, invasiveness and stemness of glioblastoma cells^8^, induce apoptosis of acute myeloid leukemia and myeloid leukemia cells^10,11^, and suppress cell proliferation and migration of bladder and gastric cancer cells^12,13^. Furthermore, PP242 also reduced the growth, proliferation and survival of colon cancer cells, and in combination with cetuximab, PP242 shows synergistic activity towards colorectal carcinoma^14,15^. However, the underlying mechanism of the effects of PP242 on the basis of the metabolome is still not clearly understood.

Metabolomics, the comprehensive study of small molecule metabolites, appears to be a robust tool to assess diverse biological samples, including cells, biofluids and tissues, in response to biological stimuli, such as disease and drugs^16,17^. Lipidomics, which is a subset of metabolomics, mainly contributes in the evaluation of various lipid species in complex biological samples^18^. Combined metabolomics and lipidomics approaches have been widely applied and used to assess the pathophysiological pathways related to disease progression, identify biomarkers and monitor the underlying mechanism of drug effects and toxicity^19^.

In this study, we examined the effects of the second-generation mTOR inhibitor PP242 in LS174T cell-induced colon cancer xenograft mouse model in order to reveal the underlying mechanism of PP242. The comprehensive metabolomics and lipidomics approaches were applied to investigate the effects of PP242 using an UHPLC-Orbitrap-MS system in mouse plasma and tumor tissues.

## Results

### Physical observations

**Figure 1:**
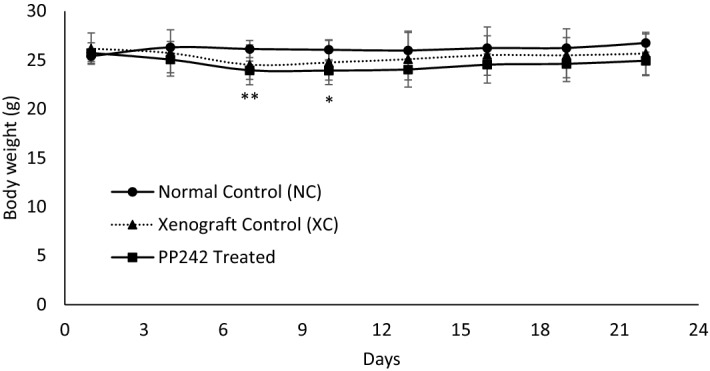
Body weight changes of normal control (NC; non tumor-bearing), xenograft control (XC; treated with vehicle), and PP242 treatment (treated with PP242 at a dose of 60 mg/kg/day) group mice for 3 weeks. *p < 0.01, **p < 0.01 [NC vs PP242 treated].

The body weights of the mice in each group (NC, normal control; XC, xenograft control; and PP242-treated) were measured once in every two days and compared (Fig. 1). Compared to the NC group, the body weights of the XC group mice decreased, but the changes were not significant. In contrast, the body weights of the PP242-treated mice decreased significantly and quickly in the first couple of days after treatment compared to the NC group, and later, the body weights gradually recovered. However, no significant change in body weight was observed between the XC and PP242-treated groups, indicating that the change in tumor size and drug treatment did not seriously affect body weight within 21 days.

**Figure 2:**
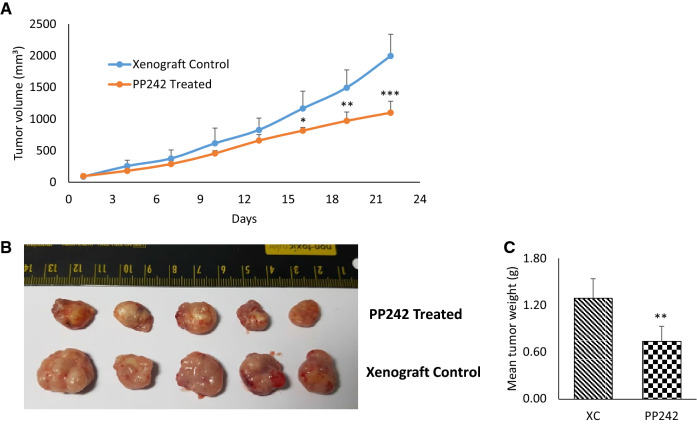
PP242 treatment showed tumor growth inhibition in vivo. (**A**) Tumor growth (in the right flank region) rate of LS174T cells induced colon cancer xenograft model after treating with vehicle (xenograft control) and PP242 (PP242 treated; 60 mg/kg/day) for 3 weeks. Treatment was started when the tumor sized reached 150–200 mm^3^. Tumor volume was presented as mean ± SD. (**B**) Representative images of tumor size of xenograft control (vehicle) and PP242 treated. (**C**) Mean weight of harvested tumor after the 3 weeks treatment of PP242. *p < 0.05; **p < 0.01; ***p < 0.001; (n = 5 per group).

Tumor-bearing mice were treated with vehicle and/or PP242 over the course of 21 days, and the size of the tumor in the right flank region was measured at two days interval using digital caliper in the LS174T xenograft groups (XC and PP242-treated) in order to evaluate the drug therapeutic effects in vivo. After 3 weeks of PP242 treatment, a very significant difference in the tumor volumes was observed (p < 0.001) before the mice were sacrificed (Fig. 2A,B). The tumors were separated after sacrifice and weighed, which further displayed the significant decrease in tumor weight for the PP242 treatment compared to the XC group mice (Fig. 2C).

### Serum biochemical parameters and histopathological tests

**Figure 3:**
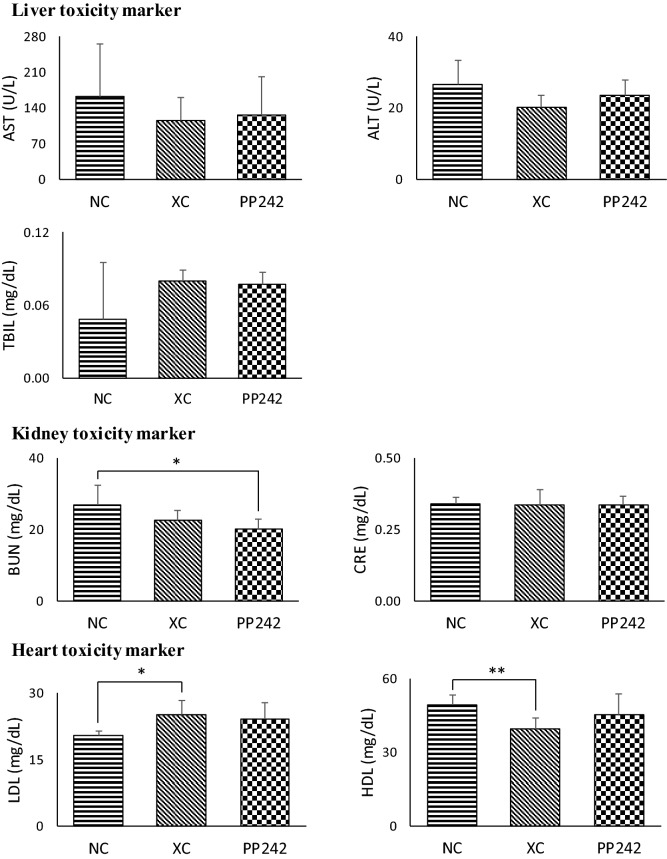
Evaluated serum biochemical parameters of normal control (NC), xenograft control (XC; vehicle treated), and PP242-treated to check the adverse effects associated with PP242. Error bar was presented as mean ± SD. *p < 0.05; **p < 0.01; ***p < 0.001; (n = 5 per group).

The results of the serum biochemical parameters test are shown in Fig. 3. All the measured serum biochemical parameters related to liver toxicity were in the normal range for all groups. No significant abnormalities were observed in the kidney toxicity biomarkers CRE and BUN either. BUN was significantly reduced only in the PP242-treated group compared to the NC group, but all were in the normal range. Alterations in cholesterol levels were observed, where LDL levels increased and HDL levels decreased in XC groups compared to NC group. However, those levels were not recovered significantly after 3 weeks of PP242 treatment.

Histological examination of the liver showed no abnormal findings in all subjects tested in the NC, XC and PP242-treated groups (Supplementary Table S1, Supplementary Fig. S1). In the histologic examination of the kidneys, no abnormal lesions were observed in any group except for cortical renal tubule hypertrophy that was observed in only one mouse among the five mouse of the PP242-treated group (Supplementary Table S1). However, it was thought that there was no kidney toxicity since the degree of hypertrophy was very minimal and the serum biochemical parameters for kidney function were normal (Supplementary Fig. S1).

Taken together, no unusual toxicological changes were observed in the liver or kidney when PP242 was administered according to the given experimental design.

### Identification of altered endogenous metabolites

**Figure 4:**
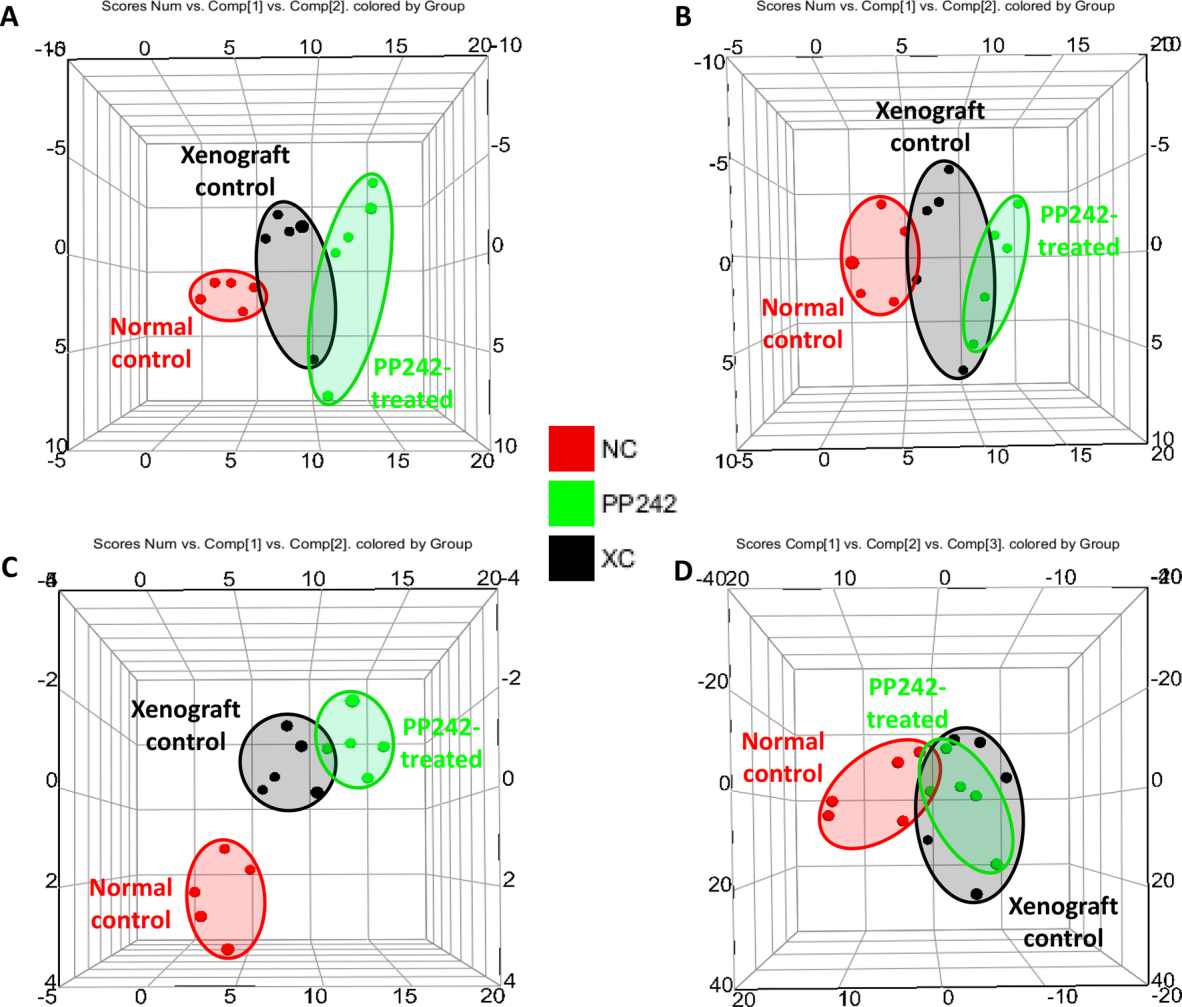
PCA score plot of plasma samples after 3 weeks treatment of PP242 at a dose of 60 mg/kg/day. (**A**) Metabolomics (ES^+^), (**B**) metabolomics (ES^−^), (**C**) Lipidomics (ES^+^), and Lipidomics (ES^−^).

**Figure 5:**
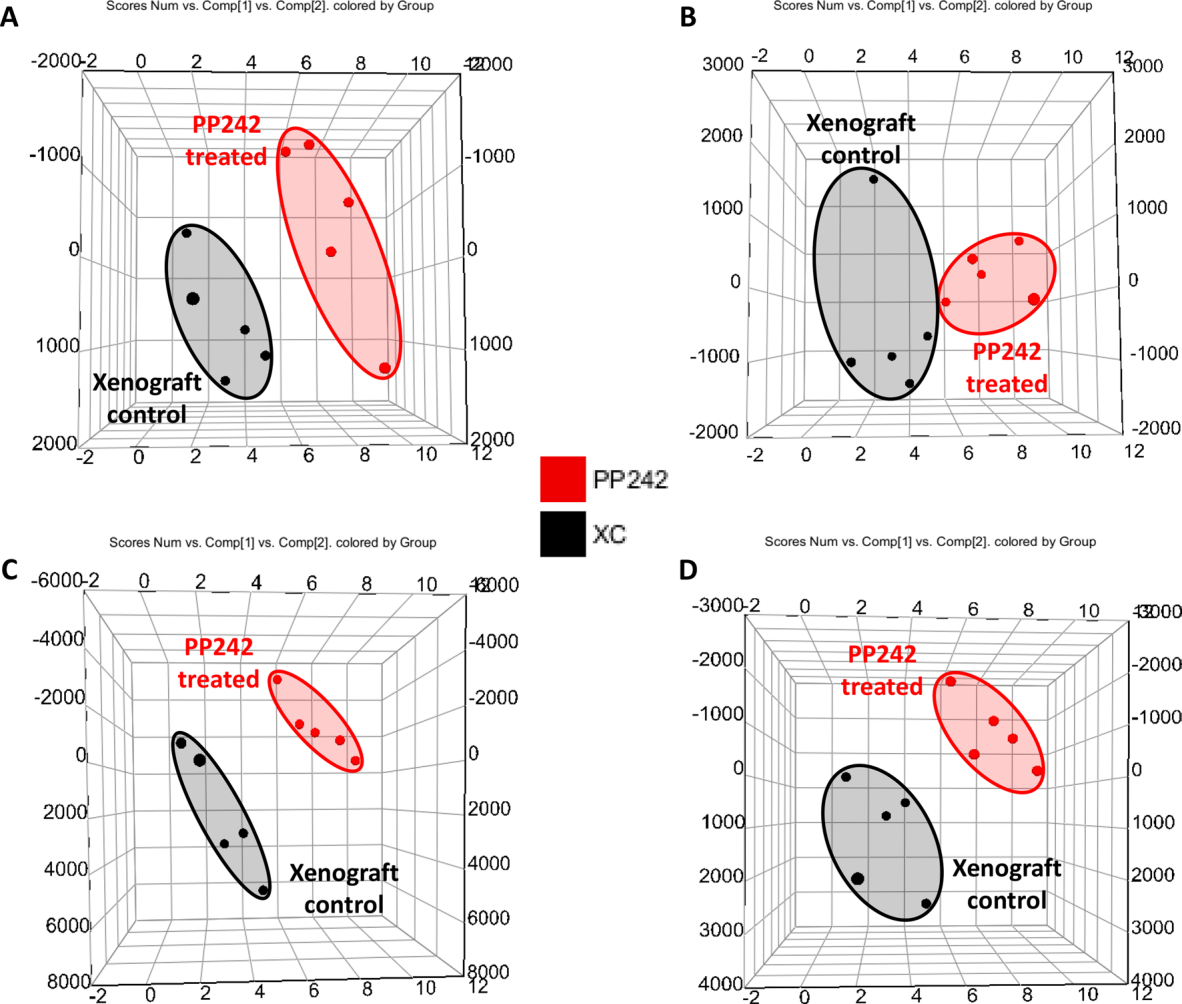
PCA score plot of tumor tissue samples after 3 weeks treatment of PP242 at a dose of 60 mg/kg/day. (**A**) Metabolomics (ES^+^), (**B**) metabolomics (ES^−^), (**C**) Lipidomics (ES^+^), and Lipidomics (ES^−^).

In order to evaluate the underlying mechanism of action of the dual mTOR inhibitor PP242, the LS174T xenograft mouse model was treated with PP242 for 3 weeks, and plasma and tumor samples were analyzed via metabolomics and lipidomics approaches. The representative base peak intensity (BPI) chromatograms of the plasma and tumor tissues for metabolomics and lipidomics studies in both positive and negative ion modes are displayed in Supplementary Figs. S2, S3. To clearly visualize the metabolic differences among the NC, XC, and PP242-treated groups in plasma and the XC and PP242-treated groups in tumor tissues, multivariate statistical analysis was used to analyze the processed mass spectrometric data. Both the principal component analysis (PCA) and partial least-squares-discriminant analysis (PLS-DA) score plots of plasma and tumor tissue metabolomics and lipidomics are shown in Figs. 4, 5, and Supplementary Figs. S4, S5 respectively. In the plasma metabolomics and lipidomics analyses, the xenograft groups (XC and PP242-treated) were clearly separated from the NC group, indicating metabolic differences due to tumor formation, but the XC and PP242-treated groups slightly overlapped (specifically in positive metabolomics; Fig. 4, Supplementary Fig. S4). In addition, both the PCA and PLS-DA score plots of tumor tissues displayed a clear separation between the XC and PP242-treated groups (Fig. 5, Supplementary Fig. S5), which clearly indicates that metabolism in tissue was altered due to the inhibition of mTOR by PP242 and that these metabolic changes were clearer in tissue than in plasma. The R^2^ and Q^2^ values for plasma metabolomics and lipidomics were in the ranges of 0.43 to 0.61 and 0.1 to 0.20, respectively, whereas those for tumor tissue were in the ranges of 0.82 to 0.99 and 0.04 to 0.87, respectively (Supplementary Fig. S5). R^2^ denotes the explanation capacity of the model, whereas Q^2^ represents the predictive ability of the model and the R^2^ and Q^2^ values close to 1 suggest an excellent model. The overall R^2^ and Q^2^ values showed that the model was reliable and had good predictability. The obtained low Q^2^ value could be because of the partial overlap of the XC and PP242 groups.

**Figure 6:**
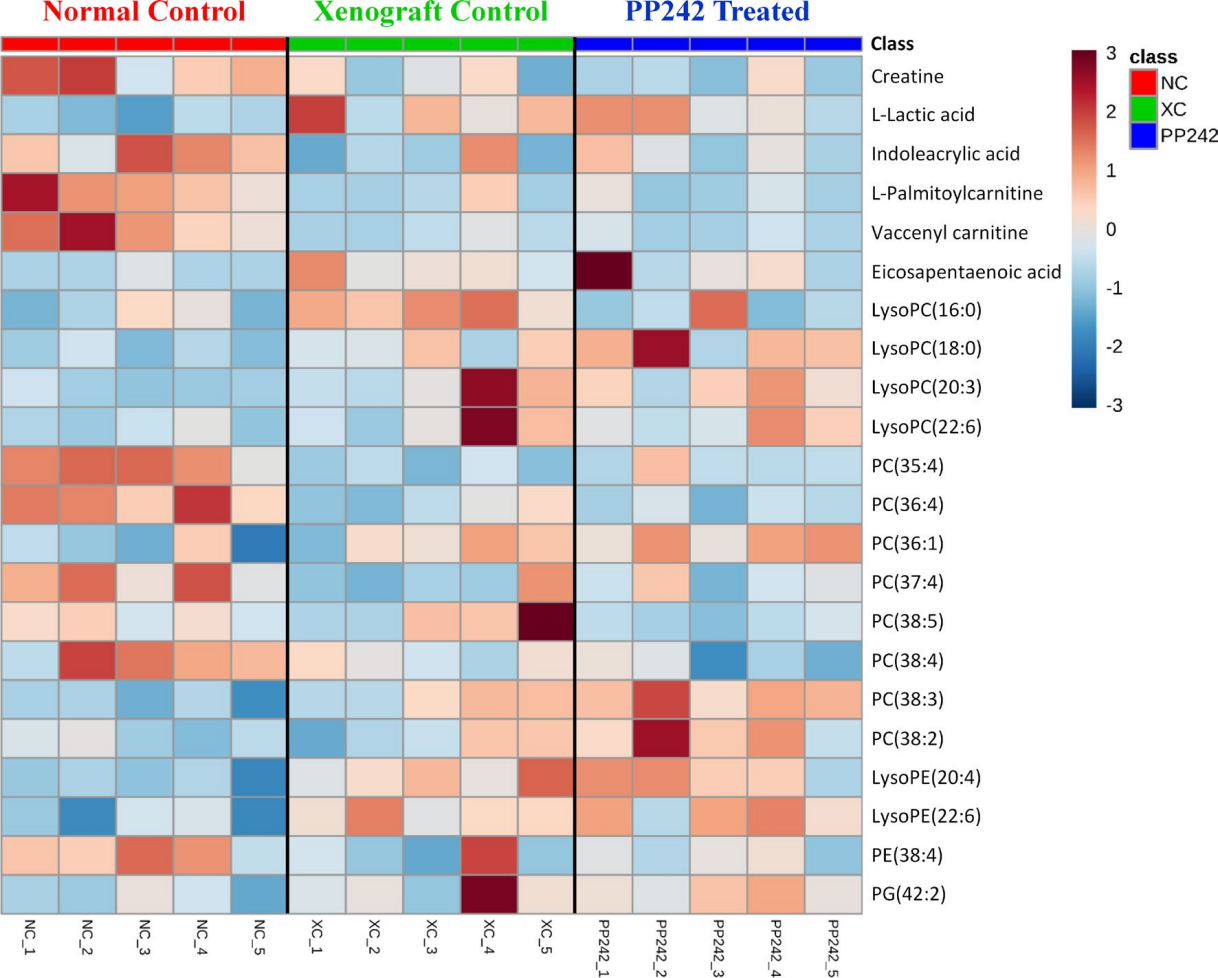
Heat map of significantly altered metabolites in plasma of normal control (NC), xenograft control (XC, tumor bearing mice treated with only vehicle), and PP242 treated (tumor bearing mice treated with PP242 at a dose of 60 mg/kg/day) groups after the 3 weeks experiment period.

In the metabolomics and lipidomics profiling of plasma, comparisons were carried out among the NC, XC and PP242 groups. The xenograft groups (XC and PP242-treated) were compared with the NC group to investigate the metabolic differences that occurred due to tumor formation and how much was recovered after PP242 treatment. Initially, a total of 49 significantly altered metabolites were identified. Of these, 22 metabolites were finally selected and used for further analyses by considering a VIP value > 1.0 and a *p* value (obtained by Student’s *t* test) < 0.05. Detailed information on the identified metabolites in plasma is listed in Table 1 and the metabolomics standards initiative (MSI) descriptions are listed in Supplementary Table S2. A heat map was also used to visualize the change pattern and is displayed in Fig. 6. The identified metabolites were mostly limited to glycerophospholipids, fatty acids and a few organic compounds. Compared to the NC group, the overall change patterns in metabolites in both xenograft groups were similar. Interestingly, none of the identified metabolites were significantly altered in the XC group compared with the PP242-treated group, and there was no consistency in the alteration pattern of all metabolite classes. Consequently, a potential effect of PP242 was not observed in plasma. However, when we observed the tumor volume and weight (Fig. 2), the changes were significant after the treatment with PP242, indicating an obvious therapeutic effect of PP242. Hence, in order to investigate the underlying meaning of the PP242 effect in tumor size reduction, we further analyzed tumor tissues.

**Table 1:** List of altered identified metabolites in plasma due to the effects of PP242 using metabolomics and lipidomics approaches.

No	Class	Metabolite Name	m/z	RT	Analytical platform	Fold change			VIP Value
						XC vs NC	PP242 vs NC	PP242 vs XC	
1	Carboxylic acids and derivatives	Creatine	132.08	0.88	Lipidomics	*0.57*	*0.48*	*0.85*	1.33
2	Hydroxy acids and derivatives	l-Lactic acid	89.02	0.93	Lipidomics	**3.32**	**2.96**	*0.89*	4.81
3	Indoles and derivatives	Indoleacrylic acid	188.07	4.83	Metabolomics	*0.56*	*0.67*	**1.18**	1.58
4	Fatty acyls	l-Palmitoylcarnitine	400.34	12.41	Metabolomics	*0.36*	*0.33*	*0.93*	1.22
5	Fatty acyls	Vaccenyl carnitine	426.36	12.59	Metabolomics	*0.21*	*0.19*	*0.9*	1.53
6	Fatty acyls	Eicosapentaenoic acid	301.22	3.42	Lipidomics	**5.58**	**6.54**	**1.17**	1.24
7	Glycerophospholipids	LysoPC(16:0)	518.32	3.82	Lipidomics	**3.05**	**1.34**	*0.44*	1.34
8	Glycerophospholipids	LysoPC(18:0)	524.37	5.37	Lipidomics	**1.29**	**1.6**	**1.24**	4.73
9	Glycerophospholipids	LysoPC(20:3)	546.35	13.67	Metabolomics	**2.48**	**2.27**	*0.92*	1.3
10	Glycerophospholipids	LysoPC(22:6)	590.32	13.28	Metabolomics	**1.92**	**1.48**	*0.77*	1.56
11	Glycerophospholipids	PC(35:4)	768.55	9.21	Lipidomics	*0.57*	*0.68*	**1.19**	1.42
12	Glycerophospholipids	PC(36:4)	782.57	8.54	Lipidomics	*0.55*	*0.51*	0.93	8.08
13	Glycerophospholipids	PC(36:1)	788.62	9.49	Lipidomics	**1.29**	**1.43**	**1.11**	2.03
14	Glycerophospholipids	PC(37:4)	796.58	8.81	Lipidomics	*0.58*	*0.65*	**1.13**	1.23
15	Glycerophospholipids	PC(38:5)	808.58	8.60	Lipidomics	**1.22**	*0.7*	*0.57*	1.89
16	Glycerophospholipids	PC(38:4)	810.60	17.17	Metabolomics	*0.79*	*0.66*	*0.83*	1.07
17	Glycerophospholipids	PC(38:3)	812.62	9.28	Lipidomics	**1.54**	**1.94**	**1.26**	3.37
18	Glycerophospholipids	PC(38:2)	814.63	9.60	Lipidomics	**1.11**	**1.52**	**1.37**	1.08
19	Glycerophospholipids	LyosPE(20:4)	500.27	13.34	Metabolomics	**1.52**	**1.54**	**1.01**	1.25
20	Glycerophospholipids	LysoPE(22:6)	524.27	13.31	Metabolomics	**1.51**	**1.58**	**1.04**	1.26
21	Glycerophospholipids	PE(38:4)	766.54	9.22	Lipidomics	*0.66*	*0.67*	1.00	1.05
22	Glycerophospholipids	PG(42:2)	857.51	7.91	Lipidomics	**1.44**	**1.42**	*0.99*	1.11

VIP value was obtained from the PLS-DA model and *p* value was calculated using Student’s *t*-test (*p < 0.05; **p < 0.01; ***p < 0.001).

Fold change > 1 represents increased metabolites (given as bold), < 1 represents decreased metabolites (given as italics).

**Figure 7:**
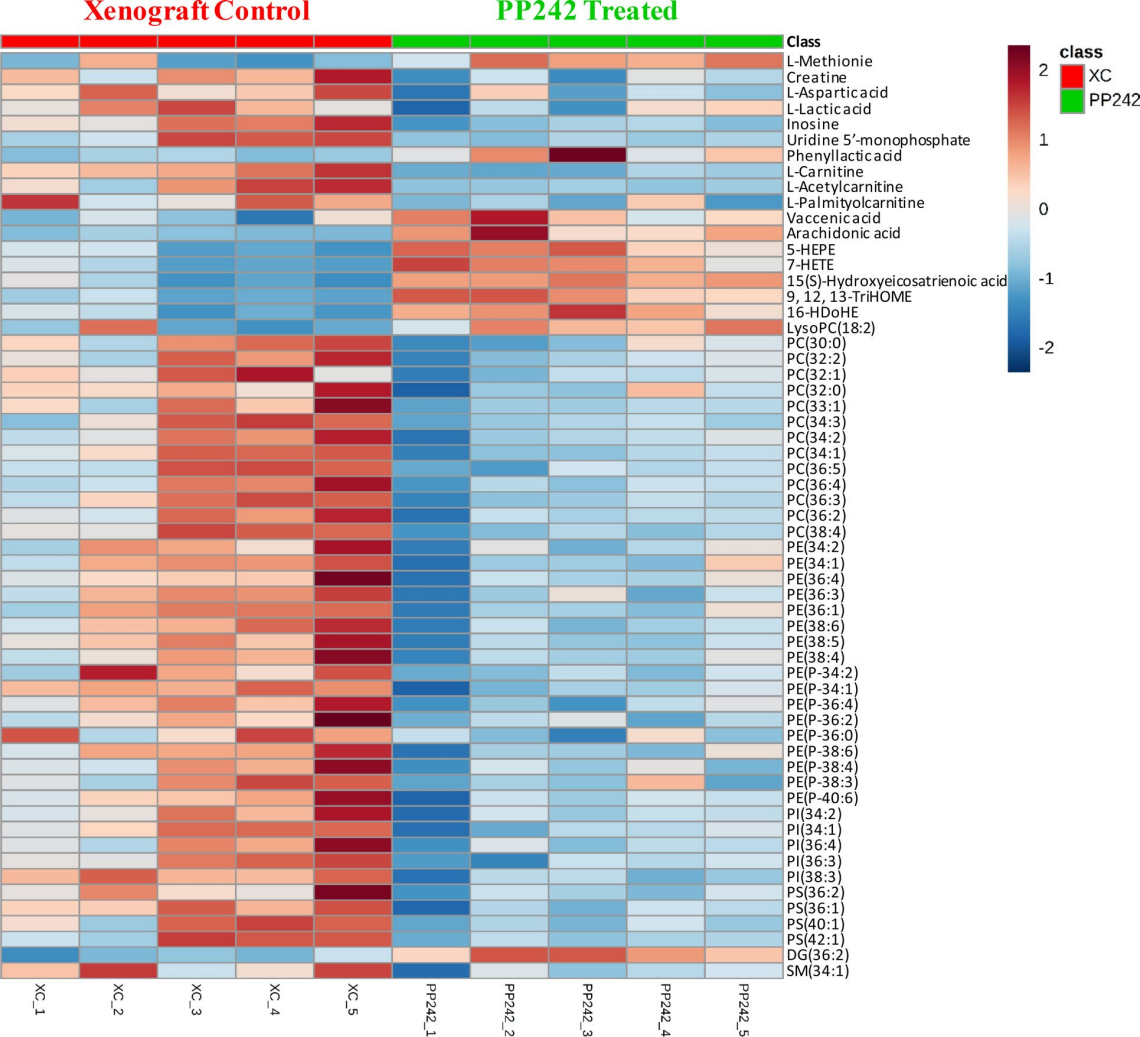
Heat map of significantly altered metabolites in tumor tissues of (XC, tumor bearing mice treated with only vehicle), and PP242 treated (tumor bearing mice treated with PP242 at a dose of 60 mg/kg/day) groups after the 3 weeks experiment period.

In tumor tissue, compared to the XC group, a total of 93 significantly altered metabolites were identified in the PP242-treated group via metabolomics and lipidomics analyses. Considering a VIP > 1.0 and a *p* value < 0.05, 59 metabolites were ultimately selected and used for further analysis (Table 2). For the ease of understanding about the metabolites identification details, MSI descriptions are also listed in Supplementary Table S3. A wide range of metabolites, including a few other organic compounds, fatty acyls, carnitines, glycerophospholipids, glycerolipids and sphingolipids, were significantly altered after PP242 treatment (Supplementary Fig. S6). We observed that the levels of all carnitines were decreased, and fatty acids, which are the precursors of various lipid molecules were increased due to the inhibition of mTOR by PP242. Other significantly altered metabolite classes were also identified, including amino acids (l-methionine), carboxylic acids and derivatives (creatine and l-aspartic acid), hydroxy acids and derivatives (l-lactic acid), purine nucleotides (inosine), pyrimidine nucleotides (uridine 5′-monophosphate) and phenylpropanoic acids (phenyllactic acid). All glycerophospholipids, including phosphatidylcholine (PC), phosphatidylethanolamine (PE), phosphatidylinositol (PI) and phosphatidylserine (PS), showed significant downregulation in the PP242-treated group compared to the XC group. However, only one identified LysoPC (LysoPC (18:2)) was significantly altered and showed upregulation after mTOR inhibition. The level of glycerolipid DG (36:2) was significantly upregulated, and the sphingolipid SM (34:1) level was significantly downregulated. A heat map is displayed in Fig. 7 to display the direct variation of each differential metabolite.

**Table 2:** List of altered identified metabolites in tumor tissues due to the effects of PP242 using metabolomics and lipidomics approaches.

No	Class	Metabolite Name	m/z	RT	Analytical platform	Fold change	VIP Value
						PP242 vs XC	
1	Amino acid	l-Methionine	150.06	1.21	**Lipidomics**	**1.7**	1.18
2	Carboxylic acids and derivatives	Creatine	132.08	0.91	Lipidomics	*0.64*	2.09
3	Carboxylic acids and derivatives	l-Aspartic acid	132.03	1.66	Lipidomics	*0.64*	1.07
4	Hydroxy acids and derivatives	l-Lactic acid	89.02	0.88	Lipidomics	*0.76*	5.29
5	Purine nucleosides	Inosine	267.07	1.50	Metabolomics	*0.37*	2.42
6	Pyrimidine nucleotides	Uridine 5′-monophosphate	323.03	1.17	Metabolomics	*0.21*	1.58
7	Phenylpropanoic acids	Phenyllactic acid	165.05	7.12	Metabolomics	**4.54**	1.03
8	Organonitrogen compounds	l-Carnitine	162.11	0.88	Lipidomics	*0.53*	1.21
9	Fatty Acyls	l-Acetylcarnitine	204.12	1.11	Metabolomics	*0.08*	1.19
10	Fatty Acyls	l-Palmitoylcarnitine	400.34	12.31	Metabolomics	*0.55*	2.31
11	Fatty Acyls	Vaccenic acid	281.25	5.41	Lipidomics	**2.03**	1.21
12	Fatty Acyls	Arachidonic acid	303.24	4.17	Lipidomics	**12.91**	1.62
13	Fatty Acyls	5-HEPE	317.21	12.11	Metabolomics	**3.63**	1.2
14	Fatty Acyls	7-HETE	319.23	12.58	Metabolomics	**3.99**	4.07
15	Fatty Acyls	15(S)-Hydroxyeicosatrienoic acid	321.24	12.81	Metabolomics	**4.08**	1.46
16	Fatty Acyls	9, 12, 13-TriHOME	329.23	10.07	Metabolomics	**4.83**	1.29
17	Fatty Acyls	16-HDoHE	343.23	12.56	Metabolomics	**3.71**	1.31
18	Glycerophospholipids	LysoPC(18:2)	520.34	13.31	Metabolomics	**2.18**	1.54
19	Glycerophospholipids	PC(30:0)	706.54	8.26	Lipidomics	*0.63*	2.88
20	Glycerophospholipids	PC(32:2)	730.54	7.92	Lipidomics	*0.52*	1.05
21	Glycerophospholipids	PC(32:1)	732.55	8.38	Lipidomics	*0.54*	3.99
22	Glycerophospholipids	PC(32:0)	734.57	8.81	Lipidomics	*0.74*	3.99
23	Glycerophospholipids	PC(33:1)	746.57	8.67	Lipidomics	*0.57*	1.28
24	Glycerophospholipids	PC(34:3)	756.55	8.21	Lipidomics	*0.39*	1.11
25	Glycerophospholipids	PC(34:2)	758.57	8.49	Lipidomics	*0.66*	5.63
26	Glycerophospholipids	PC(34:1)	760.58	8.89	Lipidomics	*0.64*	6.59
27	Glycerophospholipids	PC(36:5)	780.56	8.07	Lipidomics	*0.45*	1.04
28	Glycerophospholipids	PC(36:4)	782.57	8.42	Lipidomics	*0.62*	2.33
29	Glycerophospholipids	PC(36:3)	784.58	8.66	Lipidomics	*0.54*	3.45
30	Glycerophospholipids	PC(36:2)	786.60	9.03	Lipidomics	*0.67*	4.44
31	Glycerophospholipids	PC(38:4)	810.60	8.70	Lipidomics	*0.47*	1.61
32	Glycerophospholipids	PE(34:2)	714.51	8.65	Lipidomics	*0.74*	1.7
33	Glycerophospholipids	PE(34:1)	716.52	9.03	Lipidomics	*0.67*	1.92
34	Glycerophospholipids	PE(36:4)	738.51	8.58	Lipidomics	*0.58*	1.14
35	Glycerophospholipids	PE(36:3)	742.54	8.71	Lipidomics	*0.62*	1.1
36	Glycerophospholipids	PE(36:1)	744.55	9.52	Lipidomics	*0.73*	2.45
37	Glycerophospholipids	PE(38:6)	762.51	8.46	Lipidomics	*0.62*	1.12
38	Glycerophospholipids	PE(38:5)	764.52	8.65	Lipidomics	*0.63*	1.44
39	Glycerophospholipids	PE(38:4)	766.54	9.10	Lipidomics	*0.63*	2.47
40	Glycerophospholipids	PE(P-34:2)	698.51	8.94	Lipidomics	*0.79*	1.1
41	Glycerophospholipids	PE(P-34:1)	702.54	9.32	Lipidomics	*0.7*	1.12
42	Glycerophospholipids	PE(P-36:4)	724.53	8.87	Lipidomics	*0.7*	1.42
43	Glycerophospholipids	PE(P-36:2)	726.54	9.46	Lipidomics	*0.74*	1.29
44	Glycerophospholipids	PE(P-36:0)	730.57	9.85	Lipidomics	*0.57*	1.2
45	Glycerophospholipids	PE(P-38:6)	746.51	8.73	Lipidomics	*0.65*	2.19
46	Glycerophospholipids	PE(P-38:4)	752.56	9.38	Lipidomics	*0.7*	1.69
47	Glycerophospholipids	PE(P-38:3)	754.57	9.50	Lipidomics	*0.56*	1.23
48	Glycerophospholipids	PE(P-40:6)	776.56	9.26	Lipidomics	*0.71*	1.39
49	Glycerophospholipids	PI(34:2)	833.52	7.83	Lipidomics	*0.71*	1.6
50	Glycerophospholipids	PI(34:1)	835.53	8.21	Lipidomics	*0.69*	1.77
51	Glycerophospholipids	PI(36:4)	857.52	7.77	Lipidomics	*0.69*	1.81
52	Glycerophospholipids	PI(36:3)	859.53	7.99	Lipidomics	*0.53*	1.8
53	Glycerophospholipids	PI(38:3)	887.56	8.51	Lipidomics	*0.69*	1.75
54	Glycerophospholipids	PS(36:2)	786.53	8.42	Lipidomics	*0.74*	1.7
55	Glycerophospholipids	PS(36:1)	790.56	8.80	Lipidomics	*0.73*	1.15
56	Glycerophospholipids	PS(40:1)	844.61	9.72	Lipidomics	*0.61*	1.63
57	Glycerophospholipids	PS(42:1)	872.64	10.12	Lipidomics	*0.59*	1.47
58	Glycerolipids	DG(36:2)	621.55	17.97	Metabolomics	**3.36**	1.03
59	Sphingolipids	SM(34:1)	703.57	8.25	Lipidomics	*0.77*	2.81

VIP value was obtained from the PLS-DA model and *p* value was calculated using Student’s *t*-test (*p < 0.05; **p < 0.01; ***p < 0.001).

Fold change > 1 represents increased metabolites (given as bold), < 1 represents decreased metabolites (given as italics).

## Discussion

Mammalian target of rapamycin (mTOR) is frequently activated and overexpressed in a variety of cancers, including colon cancer. Hence, inhibition of mTOR is one of the crucial therapeutic steps in the course of cancer treatment. PP242 is an ATP-competitive inhibitor of mTOR, and the activity of PP242 in the inhibition of colon cancer growth in vitro and in vivo has been previously reported^14,20–22^. However, the underlying metabolic mechanism behind the effects of PP242 is still not clear. In the present study, using comprehensive metabolomics and lipidomics approaches, we identified that the antitumor effects of PP242 are associated with the inhibition of energy metabolism pathways, including glycolysis, the TCA cycle, fatty acid β-oxidation (β-FAO) and glycerophospholipid metabolism, in an LS174T cell-induced colon cancer xenograft mouse model.

According to the serum biochemistry and tissue histopathology test results, no unusual toxicological changes were observed in the liver and kidney when PP242 was administered at a daily dose of 60 mg/kg/day for 21 days, and these data were supported by previously published data^23^.These results mean that the change in metabolites level was not affected by the toxicity, but the effect of PP242.

Metabolomic and lipidomic approaches were performed with plasma and tumor tissue before and after treatment with PP242. In plasma, a total of 22 metabolites were identified which were significantly altered in either the XC or PP242-treated group. However, no metabolites were significantly altered between the XC and PP242-treated groups and in fact the changing patterns were inconsistent (Table 1). We observed that despite having the visible effect of PP242 in reducing tumor size (Fig. 2), changes in the plasma metabolome were not consequential to conclude the underlying mechanism.

On the other hand, 59 metabolites were significantly altered in tumor tissue after 3 weeks treatment of PP242 (Table 2), which indicates that the comprehensive metabolomics and lipidomics profiling of tumor tissues provided a better understanding and delineation of the underlying mechanism of PP242 compared to that provided by the plasma analysis.

The major perturbed metabolic pathways were mainly related to energy metabolism (glycolysis, the TCA cycle, and β-oxidation of mitochondrial fatty acids) and glycerophospholipid metabolism.

In order to maintain growth and survival, most cancer cells rely highly on glycolysis to fulfill the elevated demand for nutrients and energy, which finally leads to the elevation of lactic acid levels^24–26^. This elevation in lactic acid levels causes acidosis in the extracellular tumor microenvironment to maintain pH homeostasis and supports the migration and invasion of cancer cells^27–29^. In the present study, after PP242 treatment, the lactic acid level was significantly decreased in the tumors, indicating inhibition of glycolysis and thereby inhibiting cancer growth and invasion. We also observed a significant reduction in the level of the TCA cycle intermediate aspartic acid after PP242 treatment. Aspartic acid is the degradation metabolite of the TCA cycle, which is produced from the glutaminolysis pathway. In the glutaminolysis pathway, the production of α-ketoglutarate from glutamine via glutamic acid is another main metabolic pathway for tumor growth and survival that replenishes TCA cycle energy demand by acting as a carbon source^23,30^. Thus, the decrease in the aspartic acid level reflects the alteration of metabolites that may reduce the tumor growth of colon cancer xenograft mice by inhibiting the supply of intermediate metabolites that replenishes the energy demand of TCA cycle.

In addition to glycolysis, to meet the increased energy demand, cancer cells carry out other metabolic strategies, such as β-FAO, to produce more energy to support cancer cell growth and survival. Carnitine plays a major role in transporting long-chain fatty acids inside the inner membrane of mitochondria, which facilitates β-FAO to generate and supply acetyl-CoA to the TCA cycle for energy production^31–33^. Previous studies have reported that higher blood carnitine levels denote higher energy and functioning of cells, which is the main requirement of cancer cells, where increased levels of carnitines were reported in breast cancer and chronic lymphocytic leukemia^34,35^. According to our results, as the level of carnitines, including l-carnitine, l-acetylcarnitine, and l-palmitoylcarnitine, decreased after mTOR inhibition by PP242, the transport of fatty acids also decreased, resulting in an increase in fatty acid levels. Hence, it could be speculated that inhibition of mTOR signaling by PP242 acts by blocking β-FAO in cancer cells by reducing carnitine levels.

Treatment with PP242 also significantly reduced the glycerophospholipid (PC, PE, and PI) levels. Phosphatidylcholine (PC) is a fundamental element of the cell membrane and plays a pivotal role in the structure and function of cell membranes^36,37^. Upregulation of PC has been observed in numerous cancers, including colon cancer, and is considered one of the hallmarks of cancer growth and progression^38–40^. In this experiment, after PP242 treatment, the level of PC was significantly downregulated, suggesting that PP242 is able to inhibit cancer growth and progression. On the other hand, one LysoPC was significantly increased when the mice were exposed to PP242. LysoPC is normally generated from PC through the catalysis of phospholipase A_2_ (PLA_2_) and could increase due to the inhibition of its conversion from PC, which also decreases the level of PC^41^. Previous studies reported that the downregulation of PC and upregulation of LysoPC could trigger the induction of apoptosis^42–44^. In addition, since PP242 shows apoptotic effect on several cancer cells^10,45^, therefore, the decrease in PC and increase in LysoPC level could be due to the apoptotic effects of PP242. However, further study is required to confirm the relation of PC and LysoPC with the apoptotic effect of PP242.

Phosphatidylethanolamine (PE) is another vital element of phospholipids, and in mammalian cells, PE accounts for almost 15–25% of the total lipid content and is also associated with a vast number of physiological cellular processes^46–48^. In the normal cellular state, the existence of PE is only found in the inner leaflet of the cell membrane. However, upregulation of PE on the outer surface of cancer cells has been previously reported^47,49^. PE was significantly downregulated after PP242 treatment in our study, suggesting the potential of PP242 in treating cancer by decreasing PE levels.

We also observed a significant reduction in all phosphatidylinositol (PI) species after exposure to PP242 for 3 weeks. PI is another important phospholipid class, accounting for approximately 5.6% of total lipids, mainly exists in the inner leaflet of the cell membrane, and plays a role in regulating cell survival, signaling and membrane trafficking^50,51^. PI overexpression is also associated with cancer progression, and thus, a decrease in PI levels has shown cancer growth suppression against various cancers^52–54^. This evidence of PI level reduction supports our results and indicates the potent antitumor activity of PP242.

**Figure 8:**
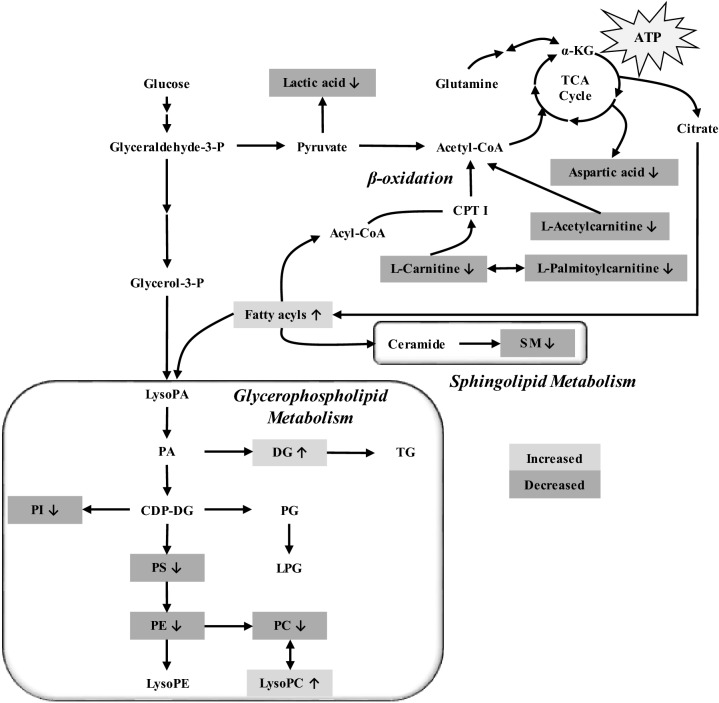
Overview of affected pathways of identified 59 significantly altered metabolites after the treatment with second generation mTOR inhibitor (PP242) at an oral dose of 60 mg/kg/day for 3 weeks. The major perturbed metabolic pathways associated with PP242 effects were energy metabolism, fatty acid metabolism and glycerophospholipid metabolism. Gray color represents increased metabolites after the mTOR inhibition by PP242; dark gray color represents decreased metabolites after the mTOR inhibition by PP242 [gray and dark gray box indicates the identified metabolites that are significantly altered].

Among the anionic phospholipids, phosphatidylserine (PS) is the most abundant and is located in the plasma membrane’s inner leaflet of most mammalian cells. It has also been reported that cancer cells possess elevated levels of PS on their external surface^55,56^. Hence, the decrease in PS levels, could suggest the suppression of cancer growth by the effects of PP242 treatment. Diacylglycerol (DG) and sphingomyelin (SM) were significantly increased and decreased, respectively. In cellular signaling and lipid metabolism, DG plays a very important role as an intermediate^35^. SM plays important roles in maintaining cell barrier functions and fluidity as a structural component of the cell membrane and regulate various cellular processes^57,58^. Additionally, depending on the tumor biology, the sphingolipid level could be increased or decreased^59^. Thus, the decrease in SM levels could express the therapeutic effects of PP242. However, the association of DG in this study is not clearly understood. A summary of the main altered metabolic and lipidomic pathways due to 3 weeks of PP242 treatment in LS174T-induced colon cancer xenografts is shown in Fig. 8.

In conclusion, herein, we observed that due to the antitumor effects of PP242, the metabolic changes were clearer in tumor tissue than in plasma of the LS174T cell-induced colon cancer xenograft mouse model. The metabolic and lipidomic investigation of tumor tissues revealed that PP242 displayed its antitumor activity by inhibiting energy and glycerophospholipid metabolism, which are the major upregulated pathways in most cancers. PP242 also exerts its anticancer effect through inhibiting the β-oxidation of fatty acids. In addition, daily doses of PP242 over 3 weeks did not induce any abnormal effects, indicating its safety level. Together, this study provides valuable insights towards understanding the underlying actions of PP242 on the basis of the metabolome and could help to further implement PP242 in clinical analysis.

## Materials and methods

### Materials

The mTOR inhibitor PP242 (torkinib, purity 97.75%) was purchased form MedChemExpress (NJ, USA). Internal standards (ISs), reserpine, and 1-Hexadecanoyl (d_31_)-2-(9Z-octadecenoyl)-sn-glycero-3-phosphocholine (PC(16:0/18:1)-d_31_) and arachidonic acid-d_8_ were purchased from Sigma-Aldrich Chemical Co. (St. Louis, MO. USA) and Avanti Polar Lipids (Alabaster, AL, USA), respectively. Formic acid, ammonium acetate and 10% neutral buffered formalin solution were purchased from the Sigma-Aldrich Chemical Co. (St. Louis, MO, USA). Ultrapure water (18.2 MΩ cm) was generated using a Milli-Q apparatus from Millipore (Milford, MA, USA). All other chemicals were of highest analytical grades and HPLC grade organic solvents were used for the preparation of mobile phases and supplied by Burdick & Jackson (SK chemicals, Ulsan, Korea).

### Cell lines

The human colon cancer cell line LS174T was purchased from American Type Culture Collection (ATCC; Manassas, VA, USA) and maintained in Dulbecco’s modified eagle’s medium (DMEM) supplemented with 10% fetal bovine serum (FBS) and 1% penicillin–streptomycin in a humidified 5% CO_2_ and 37 °C incubator.

### Development of xenograft model

All the experimental procedures were permitted by the Institutional Animal Ethics Committee of the Korea Institute of Science and Technology (KIST) and performed in accordance with the relevant guidelines and regulations (approval number: KIST-2019-005). Fifteen male BALB/c nude mice aged 6 weeks weighing 23–27 g were purchased from Nara Controls Inc. (Seoul, Korea). The mice were then housed for 1 week to acclimate to the ambient environment, and during the acclimation period, the temperature (25 ± 2 °C), the humidity (50–60%) and a 12 h light/dark cycle (8:00 am–8:00 pm) were maintained. The mice were initially divided into two groups: the normal control group (NC; n = 5) and the tumor xenograft group (n = 10). After 1 week, one million LS174T cells were subcutaneously injected into the right flank of the nude mice for xenograft model development, and blank media was injected into the normal control group following the same xenograft model procedure. Once the tumor size reached approximately 150–200 mm^3^, the xenograft group was further divided into two groups: a xenograft control group (XC; treated with vehicle, n = 5), and a PP242-treated group (treated with PP242; n = 5). PP242 solution (formulated with 2% ethanol, 20% PEG400 and 1% Tween 80 in distilled water) was administered orally to the xenograft mice at a dose of 60 mg/kg/day for 21 continuous days. The same volume of vehicle was orally administered to the NC and XC group mice for the same duration. Measurements of tumor volume in the right flank region and body weights were carried out in 2 day intervals. The tumor size was measured using a digital caliper and calculated with the formula^60^:

$$Volume = width^{2} \times {\raise0.7ex\hbox{${Length}$} \!\mathord{\left/ {\vphantom {{Length} 2}}\right.\kern-\nulldelimiterspace} \!\lower0.7ex\hbox{$2$}}$$

After 21 days of treatment, at the next day morning, the tumor size was measured. After that, the mice were anaesthetized, and blood was collected through cardiac puncture. Liver, kidney and tumor tissues were collected and immediately snap frozen in liquid nitrogen. Plasma was then obtained from the blood samples upon centrifugation. All the samples were stored at − 80 °C until further analysis.

### Serum biochemical parameters and histological analysis

In order to investigate the toxicity-related effects of PP242, serum biochemical parameters were measured. Aspartate aminotransferase (AST), alanine aminotransferase (ALT) and total bilirubin were measured to determine liver health; blood urea nitrogen (BUN) and creatinine (CRE) were measured to evaluate kidney health; and low-density lipoprotein (LDL) and high-density lipoprotein (HDL) were measured to evaluate heart health.

Histopathological examination was conducted with hepatic and renal tissues fixed in primary 10% neutral formalin solution. The left lobe of the liver and kidneys were cut to an appropriate size and thickness and fixed in the secondary formalin solution. The cut tissue was embedded in paraffin through a general tissue treatment process. Paraffin-formatted tissues were then cut to a thickness of 3 μm and subjected to hematoxylin and eosin staining, and histopathological examination was performed under an optical microscope (Olympus CX41, Japan).

### Sample preparation for metabolomics and lipidomics study

#### Plasma

For the metabolomics study, plasma samples were processed through a simple protein precipitation technique using 150 µL of ice-cold methanol using plasma methanol ratio of 1:3. After the addition of methanol, the mixture was then vortexed and centrifuged at 20,800×*g* for 10 min at 4 °C. The resulting supernatant was transferred to a new tube and diluted with water containing IS (reserpine; 4 µg/mL) at a ratio of 2:1. Finally, 5 µL of sample was injected into the UHPLC-Orbitrap-MS system after slight vortexing and spinning down. A quality control (QC) sample was made by gathering identical volumes of plasma samples and then diluting them with water containing IS (reserpine; 4 µg/mL) using the same ratio mentioned above. QC samples were used to evaluate the repeatability and robustness of the instrumental system and were analyzed before starting the sequence for column conditioning and after every ten samples in the analytical batch. Additionally, test mixtures containing a few commercially available validated standards were run at the beginning, middle and end of the analytical batch. The test mixture contained the following compounds: caffeine (0.5 µg/mL) and acetaminophen (0.5 µg/mL) for positive mode and glycocholic acid (0.5 µg/mL) and hippuric acid (0.5 µg/mL) for negative mode.

For the lipidomics study, 25 µL of PC (16:0/18:1)-d_31_ (4 µg/mL; internal standard for positive mode), 25 µL of arachidonic acid-d_8_ (4 µg/mL; internal standard for negative mode), and 50 µL of 0.1 M NaCl were added to 50 µL of the plasma samples in an Eppendorf tube. Lipid extraction was then performed by the addition of 250 µL of ice-cold chloroform/methanol (1:2; v/v) to the plasma mixture. The mixture was then vortexed for 1 min, kept at room temperature for 1 h, and centrifuged at 20,800×*g* for 10 min at 4 °C. The clear supernatant was transferred to a new tube and evaporated to dryness under a nitrogen stream at 37 °C. Finally, the dried residue was reconstituted using 60 µL of ice-cold chloroform/methanol (1:1; v/v) before being injected into the instrument for analysis. A QC sample was also prepared by gathering identical volumes from each sample after reconstitution in order to assess the repeatability and robustness of the instrument. All QC samples were run following a sequence similar to that of the metabolomics analysis.

#### Tumor tissue

The snap-frozen tumor tissues were lyophilized and homogenized into powder. Approximately 10 mg of homogenized tissue was transferred to a 2 mL Eppendorf tube, and 400 µL of methanol was added. The mixture was then sonicated for 2 min and 1 mL of MTBE (methyl tert-butyl ether) was added followed by 1 h of shaking in a shaking water bath at room temperature. Separation was induced by the addition of 250 µL of water followed by the incubation at room temperature for 10 min. After 15 min of centrifugation at 20,800×*g* at 4 °C, the upper (organic) and lower (polar) layer were separated from the precipitate and then mixed together in an another 1.5 mL Eppendorf tube. For the metabolomics study, 200 µL of mixture was taken and evaporated to dryness under a nitrogen stream at 37 °C. The dried residue was then reconstituted in 100 µL of 80% methanol, and 50 µL was loaded into the UHPLC-Orbitrap-MS system for analysis. For the tissue lipidomics study, the same volume of mixture was taken, processed following the same method and finally reconstituted in 100 µL of chloroform/methanol (2:1). In order to normalize tissue metabolomics and lipidomics data, the protein concentration of each tumor sample was quantified. Each tumor sample was diluted with distilled water, and the protein concentration was measured by Nano-MD (SINCO, Korea) using 10 µL of sample. For both the tissue metabolomics and lipidomics analyses, QC samples were made by taking identical volumes from each sample and running the samples following a sequence similar to that of plasma analysis.

### Instrumental conditions

Instrumental analysis was carried out using an Ultimate 3000 UHPLC system coupled to an LTQ Orbitrap Velos Pro mass spectrometer system (Thermo Fisher Scientific, San Jose, CA, USA) with a heated electrospray ionization (HESI) source. The same instrumental conditions and methods were used for the plasma and tumor tissue metabolomics and lipidomics analyses.

For the metabolomics analysis, an ACQUITY UPLC BEH C18 column (2.1 × 100 mm, 1.7 µm, Waters, Milford, MA, USA) was used for the chromatographic separation by maintaining the autosampler and column oven temperature at 4 °C and 50 °C, respectively. Formic acid (0.1%) in distilled water (v/v, mobile phase A) and methanol (v/v, mobile phase B) was used as the mobile phase and eluted at a flow rate of 0.4 mL/min throughout the entire analysis. The elution gradient was regulated as follows: the elution started with 100% A and was maintained for 1 min, then gradually decreased to 80% A over next 4 min, and a linear decrease of mobile phase A was made from 80 to 30% from 4 to 10 min. Mobile phase A was then decreased to 0% at 14 min followed by a rapid increase to the initial conditions for re-equilibration at the initial conditions for 2 min.

For the lipidomics analysis, chromatographic separations were performed using an ACE Excel 2 Super C18 column (2.1 × 100 mm, 1.7 µm, Advanced Chromatography Technologies Ltd., Aberdeen, Scotland, UK), and the autosampler and column oven temperature were maintained at 4 °C and 50 °C, respectively. The mobile phase was composed of 10 mM ammonium acetate in either 40% acetonitrile (v/v, mobile phase A) or acetonitrile: isopropanol (10:90, v/v, mobile phase B) and eluted at the same flow rate as that of the metabolomics study. The elution gradient was initiated using 60% A that was maintained for 1 min, and from 1 to 3 min, the concentration of A decreased to 35%. Over the next 2 min, mobile phase A decreased to 15%; then, over the next 4 min, it further decreased to 0%, and this condition was maintained for 3 min. Finally, mobile phase A was rapidly increased to reach the initial conditions for 0.5 min (60% A) and re-equilibrated for 3.5 min. The injection volume was 5 µL for all analyses.

Mass spectrometric (MS) conditions were the same for the metabolomics and lipidomics analyses of the plasma and tumor tissue samples, respectively. MS detection was operated in both positive and negative modes using a full scan ranging from *m/z* 50 to 1600, and data were acquired in centroid mode at a resolution of 60,000. High-energy collision dissociation mode was employed for the dissociation of metabolites using a normalized collision energy of 35% with an isolation width of 1 m/z and an activation time of 10 ms. The detailed MS parameters were as follows: heater temperature, 40 °C; sheath gas flow rate, 45 arb; auxiliary gas flow rate, 10 arb; spray voltage, 4 kV; capillary temperature, 320 °C; and S-lens RF level, 61%. For both the sheath gas and auxiliary gas nitrogen was used.

### Data processing and statistical analysis

After the data acquisition using LTQ-Orbitrap, the raw data were imported and processed (peak alignment, detection and identification) using the Compound Discoverer 2.1 software system. The retention time and m/z data for each peak were determined by the software. For plasma metabolomics and lipidomics analyses, all data were normalized using the peak areas of internal standards. For metabolomics and lipidomics profiling of tumor tissue samples, all data were normalized to the total protein concentrations. All multivariate data analyses were performed using SIMCA version 14.1 software (Umetrics, Inc., Ume, Sweden) system and MetaboAnalyst 4.0 (https://www.metaboanalyst.ca/). Multivariate data analyses such as principal component analysis (PCA) and partial least squares-discriminant analysis (PLS-DA) were used to visualize the separation between groups, and performed with Pareto scaling. PCA was used to visualize the general clustering among the groups, whereas PLS-DA was employed to identify the differential metabolites between groups in the plasma and tumor samples. The components with a variable importance in the projection (VIP) value exceeding 1.0 were selected as potential compounds that contributed remarkably to the clustering and showed differences between the groups. Student’s *t*-test was used to evaluate the statistical significance of each group of metabolic changes and considered significant when the value was less than 0.05.

Putative identification and searching was carried out based on the mass adducts ([M+H]^+^, [M+Na]^+^, [M−H]^−^, etc.), mass fragment (MS^2^) ions and retention time. The accuracy tolerance window of the mass was set to 10 ppm while searching the metabolites. The metabolites were searched and determined from online databases such as METLIN (https://metlin.scripps.edu/), HMDB (https://www.hmdb.ca/), lipidblast and KEGG (https://www.genome.jp/kegg) utilizing the detected m/z value and mass fragmentation patterns.

## Supplementary information

Supplementary Information.

## Data Availability

Most of the data generated during this investigation are included in this manuscript and the supplementary materials section.
