# Supplementary material for: Evaluation of the antitumor effects of PP242 in a colon cancer xenograft mouse model using comprehensive metabolomics and lipidomics
Source: Sci Rep. 2020 Oct 16;10:17523. doi: 10.1038/s41598-020-73721-w (PMC7568555; doi:10.1038/s41598-020-73721-w)
Supplement: Supplementary file 1 — Supplementary Information. [file 41598_2020_73721_MOESM1_ESM.pdf]

Supplementary Materials

Evaluation of the antitumor effects of PP242 in a colon cancer xenograft mouse model using comprehensive metabolomics and lipidomics

Md Mamunur Rashid[1,2], Hyunbeom Lee[1], Byung Hwa Jung[1,2,*]

[1]Molecular Recognition Research Center, Korea Institute of Science and Technology, Seoul 02792, Republic of Korea

[2]Division of Bio-Medical Science and Technology, KIST School, Korea University of Science and Technology (UST), Seoul 02792, Republic of Korea

***Corresponding author:**

Byung Hwa Jung, Ph.D.

Molecular Recognition Research Center, Korea Institute of Science and Technology

P.O. BOX 131, Cheongryang, Seoul 02-792, South Korea

TEL: 82-2-958-5062; FAX: 82-2-958-5059; Email: jbhluck@kist.re.kr

Supplementary Table S1. Histopathology of the liver and kidney (Summary). The parentheses indicate the percentage of the lesions. NC, Normal control; XC, Xenograft control (treated with vehicle for 21 days); PP242 (treated with PP2242 at a dose of 60 mg/kg/day for 21 days).

**Organ / Histopathology / Group**	**NC**	**XC**	**PP242**
**Liver** **No. examined**	**5**	**5**	**5**
No specific lesion	5 (100)	5 (100)	5 (100)
**Kidney** **No. examined**	**5**	**5**	**5**
No specific lesion	5 (100)	5 (100)	4 (88.0)
Hypertrophy, cortical tubule, focal	0 (0.00)	0 (0.00)	1 (20.0)

Supplementary Table S2. Metabolomics standards initiative (MSI) descriptions of the altered identified metabolites in plasma using metabolomics and lipidomics approaches.

No.	Class	Database ID	Metabolite Name	m/z	RT	Adduct	MSI identification level	MS/MS Fragments
1	Carboxylic acids and derivatives	HMDB00064	Creatine	132.08	0.88	$[M+H]^+$	level 2	90.04, 86.11
2	Hydroxy acids and derivatives	HMDB00190	L-Lactic acid	89.02	0.93	$[M-H]^-$	level 2	71.1
3	Indoles and derivatives	HMDB00734	Indoleacrylic acid	188.07	4.83	$[M+H]^+$	level 2	188.11, 170.04, 146.04
4	Fatty acyls	HMDB00222	L-Palmitoylcarnitine	400.34	12.41	$[M+H]^+$	level 2	341.27, 239.20, 144.04, 123.03
5	Fatty acyls	HMDB06351	Vaccenyl carnitine	426.36	12.59	$[M+H]^+$	level 2	408.23, 265.22, 247.20, 223.16, 144.06
6	Fatty acyls	HMDB01999	Eicosapentaenoic acid	301.22	3.42	$[M-H]^-$	level 1	301.18, 283.17, 257.23, 203.16
7	Glycerophospholipids	METLIN182	LysoPC(16:0)	518.32	3.82	$[M+Na]^+$	level 2	478.32, 184.04
8	Glycerophospholipids	HMDB10384	LysoPC(18:0)	524.37	5.37	$[M+H]^+$	level 2	506.36, 184.03
9	Glycerophospholipids	HMDB10393	LysoPC(20:3)	546.35	13.67	$[M+H]^+$	level 2	528.36, 184.01
10	Glycerophospholipids	HMDB10404	LysoPC(22:6)	590.32	13.28	$[M+Na]^+$	level 2	550.32, 184.01
11	Glycerophospholipids	HMDB07950	PC(35:4)	768.55	9.21	$[M+H]^+$	level 2	750.54, 709.49, 585.52, 544.46, 526.33, 482.33, 464.32
12	Glycerophospholipids	HMDB08138	PC(36:4)	782.57	8.54	$[M+H]^+$	level 2	782.76, 764.67, 723.55, 599.54, 520.33, 502.32
13	Glycerophospholipids	HMDB08037	PC(36:1)	788.62	9.49	$[M+H]^+$	level 2	770.63, 729.59, 605.54, 524.34, 522.43, 506.35, 504.26
14	Glycerophospholipids	NIST2769	PC(37:4)	796.58	8.81	$[M+H]^+$	level 2	778.69, 737.51, 613.47, 544.37, 526.37, 510.36, 492.35
15	Glycerophospholipids	HMDB07989	PC(38:5)	808.58	8.60	$[M+H]^+$	level 2	749.61, 625.55, 570.49, 552.36, 496.43, 478.41
16	Glycerophospholipids	HMDB08048	PC(38:4)	810.60	17.17	$[M+H]^+$	level 2	751.51, 627.53, 544.34, 524.36, 506.36
17	Glycerophospholipids	HMDB08046	PC(38:3)	812.62	9.28	$[M+H]^+$	level 2	794.63, 753.46, 629.51, 528.26, 506.39
18	Glycerophospholipids	HMDB08045	PC(38:2)	814.63	9.60	$[M+H]^+$	level 2	796.63, 755.58, 631.56, 548.39, 530.32, 524.31, 506.35
19	Glycerophospholipids	HMDB11517	LyosPE(20:4)	500.27	13.34	$[M-H]^-$	level 2	303.22, 214.01
20	Glycerophospholipids	HMDB11526	LysoPE(22:6)	524.27	13.31	$[M-H]^-$	level 2	327.20, 214.05, 196.12
21	Glycerophospholipids	HMDB09387	PE(38:4)	766.54	9.22	$[M-H]^-$	level 2	500.31, 482.32, 480.31, 462.38, 303.25, 283.26
22	Glycerophospholipids	NIST113518	PG(42:2)	857.51	7.91	$[M-H]^-$	level 2	619.44, 601.37, 391.27, 255.14

Supplementary Table S3. Metabolomics standards initiative (MSI) descriptions of the altered identified metabolites in tumor using metabolomics and lipidomics approaches.

No.	Class	Database ID	Metabolite Name	m/z	RT	Adduct	MSI identification level	MS/MS Fragments
1	Amino acid	HMDB00696	L-Methionine	150.06	1.21	$[M+H]^+$	level 2	132.97, 104.00, 102.00, 56.03
2	Carboxylic acids and derivatives	HMDB00064	Creatine	132.08	0.91	$[M+H]^+$	level 2	132.02, 114.01, 90.01
3	Carboxylic acids and derivatives	HMDB00191	L-Aspartic acid	132.03	1.66	$[M-H]^-$	level 2	131.98, 114.96, 88.09
4	Hydroxy acids and derivatives	HMDB00190	L-Lactic acid	89.02	0.88	$[M-H]^-$	level 2	71.1
5	Purine nucleosides	HMDB00195	Inosine	267.07	1.50	$[M-H]^-$	level 2	249.08, 176.98, 134.95
6	Pyrimidine nucleotides	HMDB00288	Uridine 5'-monophosphate	323.03	1.17	$[M-H]^-$	level 2	322.93, 210.93, 97.02
7	Phenylpropanoic acids	HMDB00779	Phenyllactic acid	165.05	7.12	$[M-H]^-$	level 2	165.0, 147.0, 119.06, 103.06, 73.02
8	Organonitrogen compounds	HMDB00062	L-Carnitine	162.11	0.88	$[M+H]^+$	level 2	103.03, 85.05, 60.11
9	Fatty Acyls	HMDB00201	L-Acetylcarnitine	204.12	1.11	$[M+H]^+$	level 2	145.03, 85.05, 60.11
10	Fatty Acyls	HMDB00222	L-Palmitoylcarnitine	400.34	12.31	$[M+H]^+$	level 2	341.20, 239.15, 144.14
11	Fatty Acyls	HMDB03231	Vaccenic acid	281.25	5.41	$[M-H]^-$	level 2	281.27, 263.22, 237.25
12	Fatty Acyls	HMDB01043	Arachidonic acid	303.24	4.17	$[M-H]^-$	level 1	303.23, 285.18, 259.26, 205.15
13	Fatty Acyls	HMDB05081	5-HEPE	317.21	12.11	$[M-H]^-$	level 2	317.24, 299.21, 281.26, 273.30, 255.21, 201.21
14	Fatty Acyls	HMDB62431	7-HETE (7-Hydroxyeicosatetraenoic acid)	319.23	12.58	$[M-H]^-$	level 2	319.24, 301.23, 283.20, 275.20, 257.22, 221.27, 207.16
15	Fatty Acyls	HMDB05045	15(S)-Hydroxyeicosatrienoic acid	321.24	12.81	$[M-H]^-$	level 2	321.22, 303.19, 285.27, 277.20, 259.19, 221.14
16	Fatty Acyls	HMDB04708	9, 12, 13-TriHOME	329.23	10.07	$[M-H]^-$	level 2	329.22, 311.24, 293.21, 285.16, 267.22, 229.13, 211.10
17	Fatty Acyls	HMDB60047	16-HDoHE	343.23	12.56	$[M-H]^-$	level 2	343.25, 325.24, 299.26, 281.26, 261.21, 245.21, 233.15
18	Glycerophospholipids	HMDB10386	LysoPC(18:2)	520.34	13.31	$[M+H]^+$	level 2	502.27, 184.0
19	Glycerophospholipids	HMDB07869	PC(30:0)	706.54	8.26	$[M+H]^+$	level 2	706.54, 688.60, 647.52, 523.55, 496.33, 478.34, 468.31, 450.28
20	Glycerophospholipids	HMDB07874	PC(32:2)	730.54	7.92	$[M+H]^+$	level 2	730.57, 712.66, 671.49, 547.49, 520.35, 502.34, 468.34, 450.27
21	Glycerophospholipids	HMDB07872	PC(32:1)	732.55	8.38	$[M+H]^+$	level 2	714.60, 673.66, 549.54, 522.29, 504.39, 468.34, 450.30
22	Glycerophospholipids	HMDB00564	PC(32:0)	734.57	8.81	$[M+H]^+$	level 2	734.59, 716.48, 675.60, 551.61, 496.30, 478.29

23	Glycerophospholipids	HMDB07939	PC(33:1)	746.57	8.67	$[M+H]^+$	level 2	746.68, 728.59, 687.55, 522.38, 504.41, 482.31, 464.33
24	Glycerophospholipids	HMDB08006	PC(34:3)	756.55	8.21	$[M+H]^+$	level 2	757.59, 738.66, 697.45, 573.58, 571.51, 520.39, 502.40, 494.32, 476.35
25	Glycerophospholipids	HMDB08004	PC(34:2)	758.57	8.49	$[M+H]^+$	level 2	758.65, 740.60, 699.53, 575.56, 522.35, 504.35, 494.34, 476.36
26	Glycerophospholipids	HMDB07971	PC(34:1)	760.58	8.89	$[M+H]^+$	level 2	742.51, 701.61, 577.51, 522.30, 504.34, 496.41, 478.30
27	Glycerophospholipids	HMDB07984	PC(36:5)	780.56	8.07	$[M+H]^+$	level 2	780.67, 762.66, 721.52, 597.52, 542.44, 524.34, 496.38, 478.37
28	Glycerophospholipids	HMDB08138	PC(36:4)	782.57	8.42	$[M+H]^+$	level 2	764.52, 723.52, 599.42, 520.27, 502.29
29	Glycerophospholipids	HMDB08105	PC(36:3)	784.58	8.66	$[M+H]^+$	level 2	784.66, 766.74, 725.47, 601.53, 522.34, 520.36, 504.45, 502.34
30	Glycerophospholipids	HMDB00593	PC(36:2)	786.60	9.03	$[M+H]^+$	level 2	768.62, 727.52, 603.66, 522.34, 504.37
31	Glycerophospholipids	HMDB08145	PC(38:4)	810.60	8.70	$[M+H]^+$	level 2	810.73, 792.69, 757.58, 627.59, 548.40, 530.33, 502.33
32	Glycerophospholipids	HMDB08928	PE(34:2)	714.51	8.65	$[M-H]^-$	level 2	476.23, 458.24, 452.29, 434.31, 279.22, 255.23
33	Glycerophospholipids	HMDB08926	PE(34:1)	716.52	9.03	$[M-H]^-$	level 2	478.25, 460.22, 452.27, 434.38, 281.22, 255.18
34	Glycerophospholipids	HMDB08938	PE(36:4)	738.51	8.58	$[M-H]^-$	level 2	740.56, 722.65, 599.41, 502.28, 484.27, 454.36, 361.32, 313.22
35	Glycerophospholipids	HMDB09319	PE(36:3)	742.54	8.71	$[M+H]^+$	level 2	601.48, 486.43, 436.39
36	Glycerophospholipids	HMDB08992	PE(36:1)	744.55	9.52	$[M-H]^-$	level 2	480.33, 478.29, 462.32, 460.38, 283.24, 281.24
37	Glycerophospholipids	HMDB08946	PE(38:6)	762.51	8.46	$[M-H]^-$	level 2	524.29, 506.33, 452.28, 434.30, 327.23, 255.25
38	Glycerophospholipids	HMDB09036	PE(38:5)	764.52	8.65	$[M-H]^-$	level 2	500.32, 482.22, 478.24, 460.23, 303.17, 281.20
39	Glycerophospholipids	HMDB09387	PE(38:4)	766.54	9.10	$[M-H]^-$	level 2	500.31, 482.32, 480.31, 462.38, 303.25, 283.26
40	Glycerophospholipids	HMDB11343	PE(P-34:2)	698.51	8.94	$[M-H]^-$	level 2	436.26, 418.22, 279.17
41	Glycerophospholipids	HMDB08952	PE(P-34:1)	702.54	9.32	$[M+H]^+$	level 2	684.57, 561.53, 364.28, 339.30, 266.30
42	Glycerophospholipids	HMDB11353	PE(P-36:4)	724.53	8.87	$[M+H]^+$	level 2	706.55, 583.50, 364.29, 266.29
43	Glycerophospholipids	HMDB11376	PE(P-36:2)	726.54	9.46	$[M-H]^-$	level 2	464.37, 446.35, 279.25
44	Glycerophospholipids	HMDB09016	PE(P-36:0)	730.57	9.85	$[M-H]^-$	level 2	730.62, 712.47, 684.46, 464.31, 446.30, 283.22
45	Glycerophospholipids	HMDB09708	PE(P-38:6)	746.51	8.73	$[M-H]^-$	level 2	506.24, 436.26, 418.26, 375.20, 327.20, 309.24, 283.22
46	Glycerophospholipids	HMDB11358	PE(P-38:4)	752.56	9.38	$[M+H]^+$	level 2	734.56, 611.55, 389.28, 364.27, 266.27
47	Glycerophospholipids	HMDB11384	PE(P-38:3)	754.57	9.50	$[M+H]^+$	level 2	736.59, 613.57, 392.32, 363.31, 294.31
48	Glycerophospholipids	HMDB11394	PE(P-40:6)	776.56	9.26	$[M+H]^+$	level 2	758.56, 635.52, 392.31, 294.34
49	Glycerophospholipids	HMDB09801	PI(34:2)	833.52	7.83	$[M-H]^-$	level 2	597.40, 579.31, 569.36, 551.32, 417.22, 389.19, 281.22, 253.28
50	Glycerophospholipids	HMDB09783	PI(34:1)	835.53	8.21	$[M-H]^-$	level 2	597.36, 579.23, 571.27, 553.26, 417.20, 391.16, 281.17, 255.18
51	Glycerophospholipids	HMDB09790	PI(36:4)	857.52	7.77	$[M-H]^-$	level 2	619.32, 601.32, 571.32, 553.31, 439.25, 391.23, 303.24, 255.22

52	Glycerophospholipids	HMDB09826	PI(36:3)	859.53	7.99	$[M-H]^-$	level 2	597.28, 595.26, 579.29, 577.30, 417.22, 415.19, 281.22, 279.18
53	Glycerophospholipids	HMDB09813	PI(38:3)	887.56	8.51	$[M-H]^-$	level 2	621.39, 603.34, 599.35, 581.32, 441.20, 419.26, 305.25, 283.28
54	Glycerophospholipids	HMDB12400	PS(36:2)	786.53	8.42	$[M-H]^-$	level 2	699.50, 437.23, 433.19, 419.20, 415.22, 283.20, 279.19
55	Glycerophospholipids	HMDB112289	PS(36:1)	790.56	8.80	$[M+H]^+$	level 2	703.52, 605.53, 526.29, 524.37, 508.30, 506.25
56	Glycerophospholipids	HMDB0112417	PS(40:1)	844.61	9.72	$[M-H]^-$	level 2	757.61, 493.29, 475.30, 435.24, 417.22, 339.30, 281.23
57	Glycerophospholipids	HMDB0112407	PS(42:1)	872.64	10.12	$[M-H]^-$	level 2	785.65, 521.44, 503.38, 435.34, 417.25, 367.38, 281.27
58	Glycerolipids	HMDB07188	DG(36:2)	621.55	17.97	$[M+NH4]^+$	level 2	621.50, 603.56, 339.28
59	Sphingolipids	HMDB10169	SM(34:1)	703.57	8.25	$[M+H]^+$	level 2	703.59, 685.57, 644.55, 626.46, 520.56

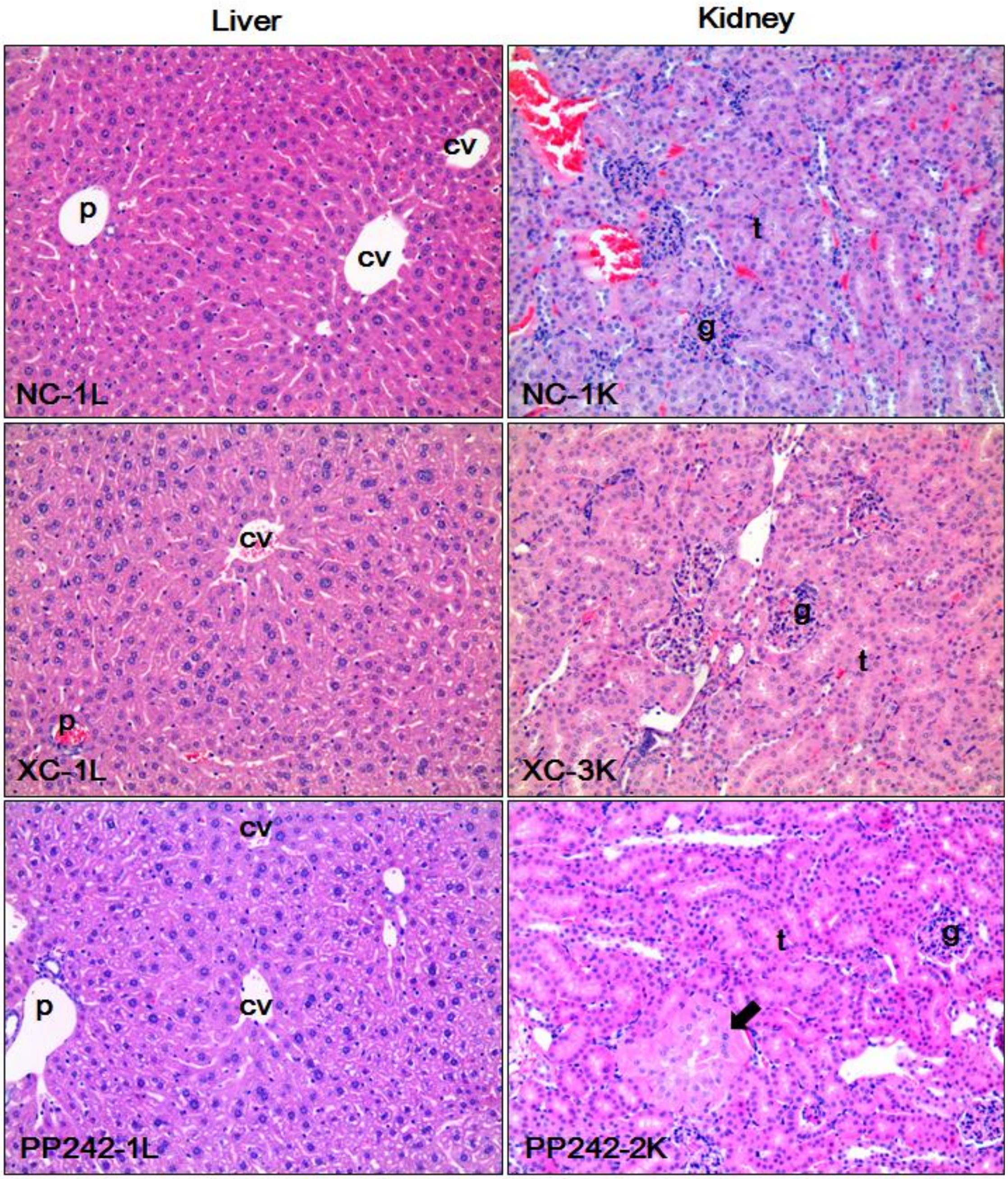

Supplementary Figure S1. Histological features of the representative liver and kidney after three weeks of PP242 treatment at a dose of 60 mg/kg/day. No specific treatment-related abnormal findings were noted in the liver and kidney. In the PP242-2K, note the hypertrophic renal tubule (thick arrow) in the cortex. p, portal triad; cv, central vein; g, glomerulus; t, renal tubule. H&E. Mag.=×200 for all.

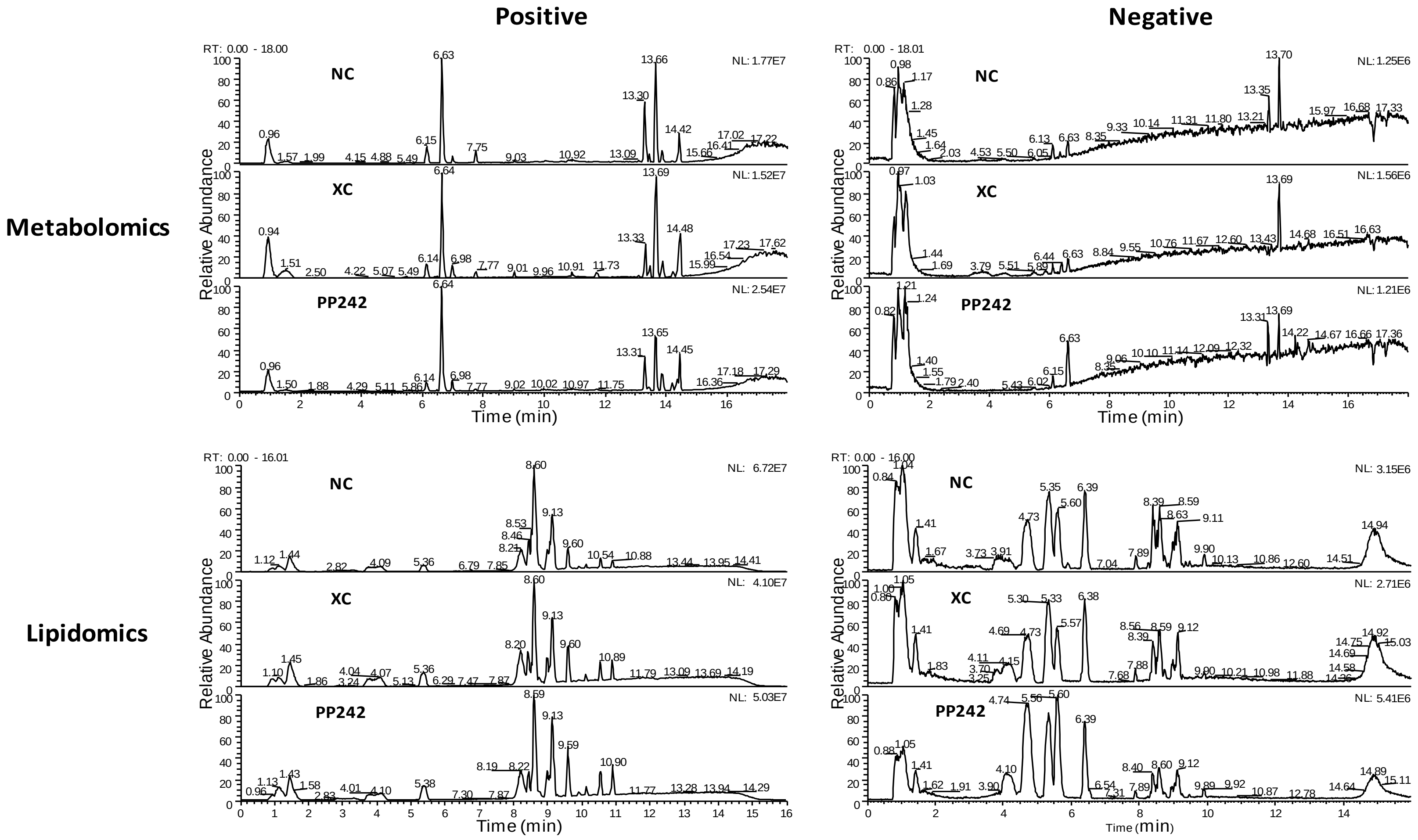

Supplementary Figure S2. Representative base peak intensity (BPI) chromatograms of plasma metabolomics and lipidomics samples of normal control (NC), xenograft control (tumor bearing mice treated with only vehicle) and PP242-treated (tumor bearing mice treated with PP242 at a dose of 60 mg/kg/day) groups.

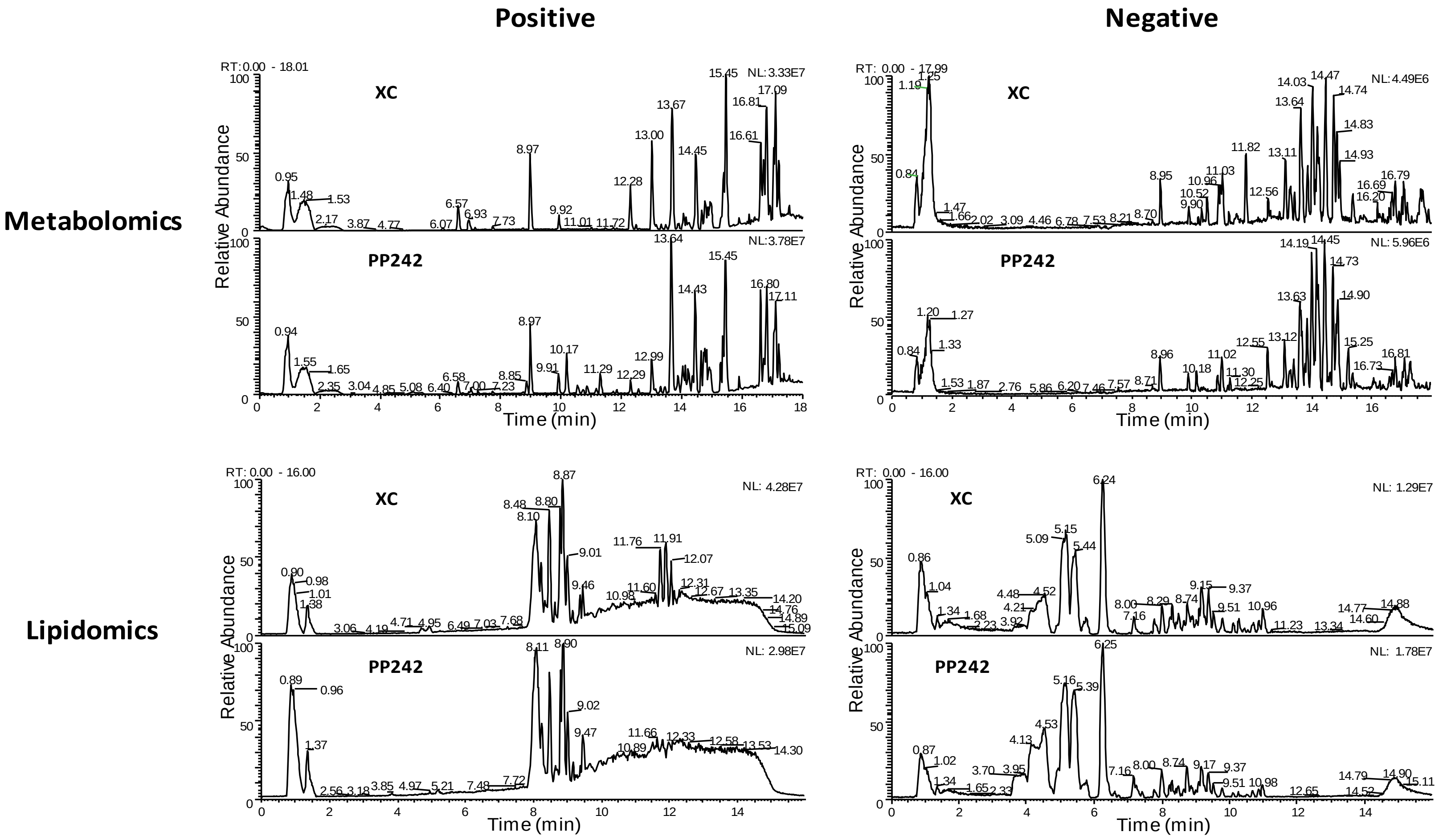

Supplementary Figure S3. Representative base peak intensity (BPI) chromatograms of tumor metabolomics and lipidomics samples of xenograft control (tumor bearing mice treated with only vehicle) and PP242-treated (tumor bearing mice treated with PP242 at a dose of 60 mg/kg/day) groups.

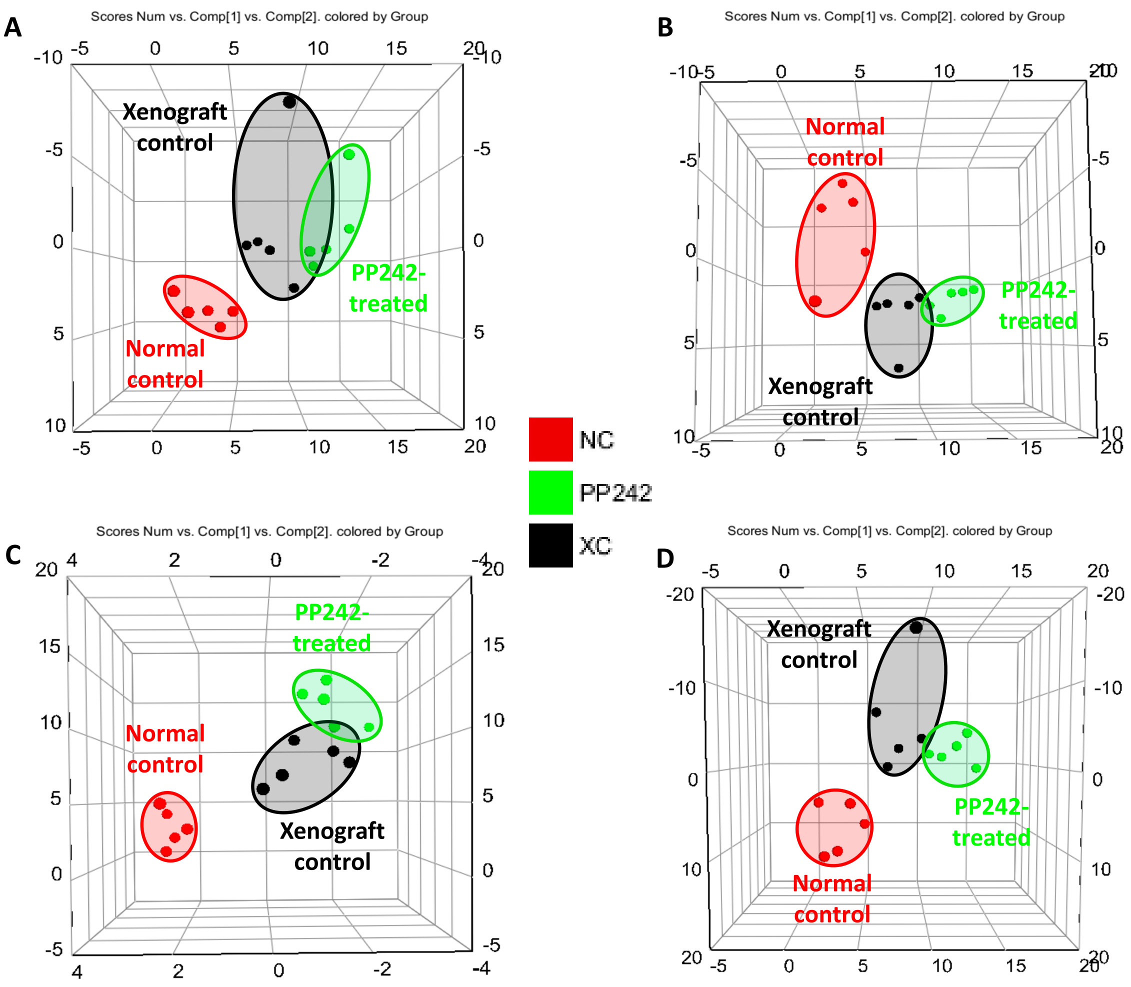

Supplementary Figure S4. PLS-DA score plot of plasma samples after three weeks treatment of PP242 at a dose of 60 mg/kg/day. (A) Metabolomics (ES^+; $R^2 = 0.43$, $Q^2 = 0.1$), (B) metabolomics (ES^-; $R^2 = 0.43$, $Q^2 = 0.21$), (C) Lipidomics (ES^+; $R^2 = 0.61$, $Q^2 = 0.20$), and Lipidomics (ES^-; $R^2 = 0.51$, $Q^2 = 0.17$).

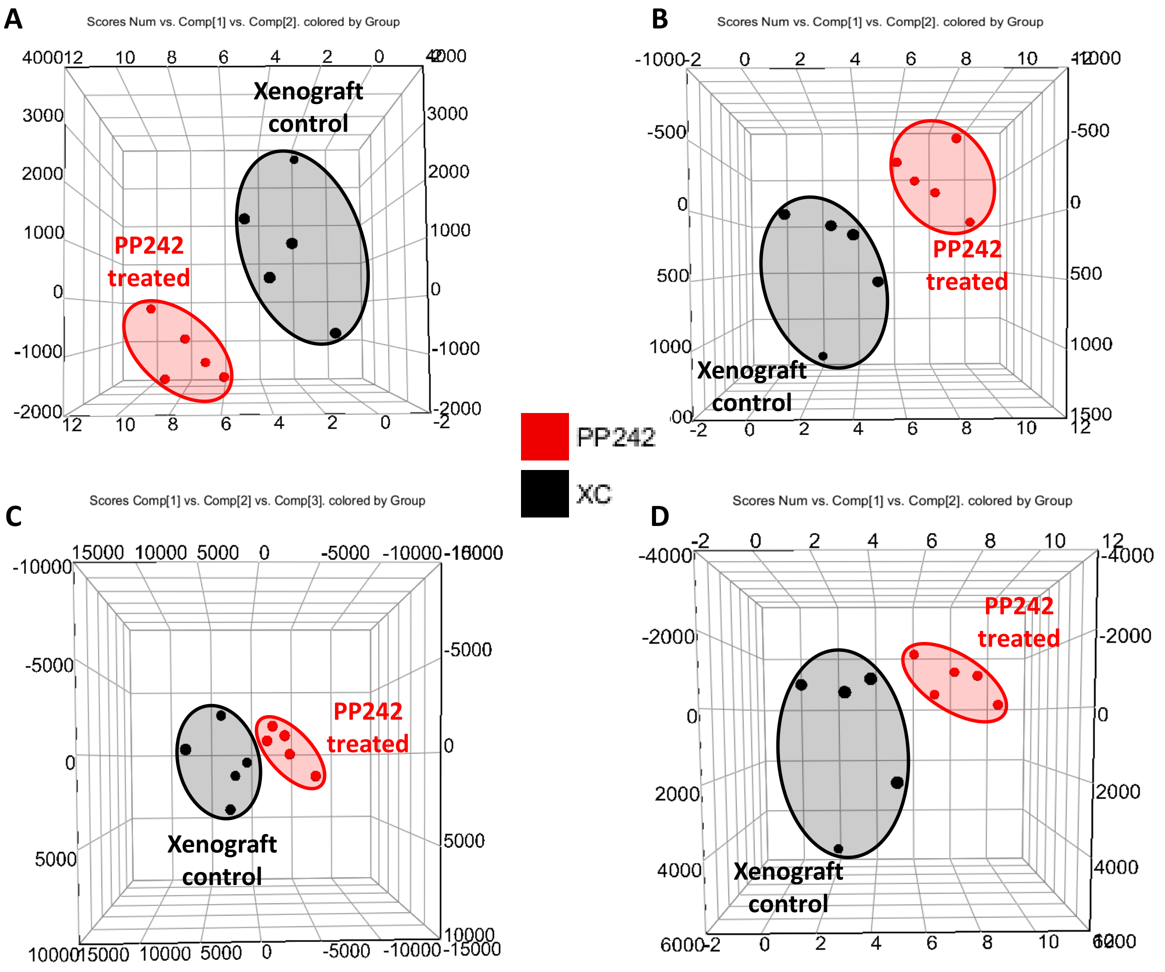

Supplementary Figure S5. PLS-DA score plot of tumor tissue samples after three weeks treatment of PP242 at a dose of 60 mg/kg/day. (A) Metabolomics (ES^+; $R^2 = 0.89$, $Q^2 = 0.60$), (B) metabolomics (ES^-; $R^2 = 0.94$, $Q^2 = 0.52$), (C) Lipidomics (ES^+; $R^2 = 0.99$, $Q^2 = 0.87$), and Lipidomics (ES^-; $R^2 = 0.82$, $Q^2 = 0.04$).

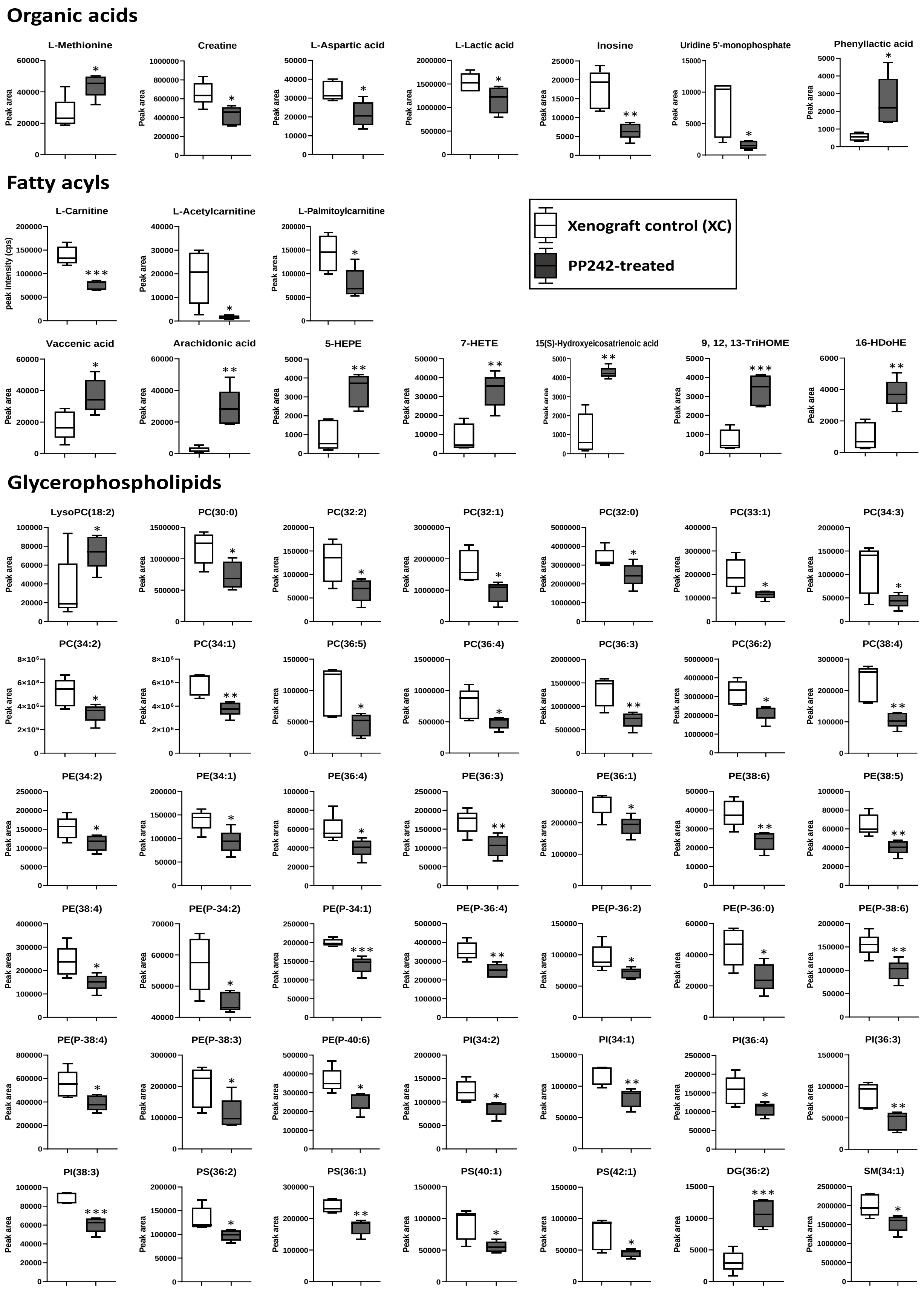
Organic acids
L-Methionine
Creatine
L-Aspartic acid
L-Lactic acid
Inosine
Uridine 5'-monophosphate
Phenyllactic acid
Fatty acyls
L-Carnitine
L-Acetylcarnitine
L-Palmitoylcarnitine
Xenograft control (XC)
PP242-treated
Vaccenic acid
Arachidonic acid
5-HEPE
7-HETE
15(S)-Hydroxyeicosatrienoic acid
9, 12, 13-TriHOME
16-HDoHE
Glycerophospholipids
LysoPC(18:2)
PC(30:0)
PC(32:2)
PC(32:1)
PC(32:0)
PC(33:1)
PC(34:3)
PC(34:2)
PC(34:1)
PC(36:5)
PC(36:4)
PC(36:3)
PC(36:2)
PC(38:4)
PE(34:2)
PE(34:1)
PE(36:4)
PE(36:3)
PE(36:1)
PE(38:6)
PE(38:5)
PE(38:4)
PE(P-34:2)
PE(P-34:1)
PE(P-36:4)
PE(P-36:2)
PE(P-36:0)
PE(P-38:6)
PE(P-38:4)
PE(P-38:3)
PE(P-40:6)
PI(34:2)
PI(34:1)
PI(36:4)
PI(36:3)
PI(38:3)
PS(36:2)
PS(36:1)
PS(40:1)
PS(42:1)
DG(36:2)
SM(34:1)
Peak area
peak intensity (cps)

Supplementary Fig S6. Box-and-whisker plot of identified significantly altered metabolites in tumor tissues. The *p* value was calculated using Student's t-test, *$p<0.05$; **$p<0.01$; ***$p<0.001$ (XC vs PP242-treated).
